# Design of field trials for the evaluation of transmissible vaccines in animal populations

**DOI:** 10.1371/journal.pcbi.1012779

**Published:** 2025-02-03

**Authors:** Justin K. Sheen, Lee Kennedy-Shaffer, Michael Z. Levy, Charlotte Jessica E. Metcalf

**Affiliations:** 1 Department of Ecology and Evolutionary Biology, Princeton University, Princeton, New Jersey, United States of America; 2 Department of Mathematics and Statistics, Vassar College, Poughkeepsie, New York, United States of America; 3 Department of Biostatistics, Yale School of Public Health, New Haven, Connecticut, United States of America; 4 Department of Biostatistics, Epidemiology and Informatics, University of Pennsylvania, Philadelphia, Pennsylvania, United States of America; 5 School of Public and International Affairs, Princeton University, Princeton, New Jersey, United States of America; Fundação Getúlio Vargas: Fundacao Getulio Vargas, BRAZIL

## Abstract

Vaccines which can transmit from vaccinated to unvaccinated animals may be especially useful for increasing immunity in hard to reach populations or in populations where achieving high coverage is logistically infeasible. However, gauging the public health utility for future use of such transmissible vaccines and assessing their risk-benefit tradeoff, given their potential for unintended evolution, hinges on accurate estimates of their indirect protective effect. Here, we establish the conditions under which a two-stage randomized field trial can characterize the protective effects of a transmissible vaccine relative to a traditional vaccine. We contrast the sample sizes required to adequately power these trials when the vaccine is weakly and strongly transmissible. We also identify how required sample sizes change based on the characteristics of host ecology such as the overdispersion of the contact structure of the population, as well as the efficacy of the vaccine and timing of vaccination. Our results indicate the range of scenarios where two-stage randomized field trial designs are feasible and appropriate to capture the protective effects of transmissible vaccines. Our estimates identify the protective benefit of using transmissible vaccines compared to traditional vaccines, and thus can be used to weigh against evolutionary risks.

## Author summary

Transmissible vaccines, which are able to spread from vaccinated animals to unvaccinated animals, have the potential to vaccinate a larger proportion of animal populations than a traditional, non-transmissible vaccine. However, prior to deployment, investigators need methods that characterize these unique protective effects, such as protection via the indirect vaccination of initially unvaccinated individuals. In this work we propose a two-stage randomized trial design to evaluate transmissible vaccines and define causal estimands that capture protections conferred by a transmissible vaccine and statistical estimands that capture other features of the population impacts. Through simulation we show that these trials can identify the protective benefits of transmissible vaccines relative to traditional vaccines, and that such trials are logistically feasible for many animal populations. We also develop an analytical solution that can quickly estimate the approximate sample size required to adequately power such a trial under a variety of characteristics of the vaccine and host ecology, such as the transmissibility of the vaccine and contact structure of the host.

## Introduction

Characterizing how the benefits of vaccinating one individual scales across the population is an important question in the management of pathogens that circulate in livestock and wildlife animal populations. Standard, non-transmissible vaccines directly protect vaccinated animals by reducing mortality and morbidity, and may also have secondary/indirect protective effects for the contacts of the vaccinated animal by blocking transmission chains [1-3]. Transmissible vaccines, where the vaccines may self-replicate and transmit to unvaccinated animals from a vaccinated animal within their social cluster, should provide even greater population-level protection per animal vaccinated than standard vaccines. Transmissible vaccines include live-attenuated viruses, which are genetically weakened forms of wild-type pathogens, and recombinant vector vaccines (RVV), which are harmless viruses with genetic insertions of parts of the wild-type pathogens [4]. While the potential level of protection that these vaccines might induce in a population has been the subject of much investigation [4-6], less work has been done on how to quantitatively assess these predictions in field conditions. Importantly, transmissibility of such vaccines is no guarantee of high indirect protection from disease; for example, if the population has a sparse contact structure, then the vaccine may not be able to transmit effectively in the population, although the wildtype pathogen may still circulate. Understanding whether or not transmissible vaccines deliver the benefits they promise has relevance for optimal resource use, but also in weighing evolutionary concerns. Although strongly transmissible vaccines will be more effective at reaching animals, more transmission also provides more opportunities for mutation and selection for problematic vaccine variants, e.g., pathogenic variants. Reliable estimates of the exact benefits of transmissible vaccines are required to weigh against these risks [6-10].

Over the past decade, the potential utility of these self-disseminating vaccines has led to their proposed deployment for a wide spectrum of pathogens that circulate in both livestock and wildlife animal populations [11-13]. Transmissible vaccines that target poultry viruses such as avian influenza may be especially useful given the potential for zoonotic spillover [11]. Development of transmissible vaccines against rabies has also been proposed for hard-to-vaccinate vampire bat populations [12]. The value of transmissible vaccines will be defined by the degree of indirect protection that they confer, in addition to the direct protection of vaccinated animals. Even weakly transmissible vaccines for animal populations—defined here as vaccines with a reproductive value R_0,v_ < 1, meaning that a vaccinated individual on average transmits the vaccine to less than one other individual—have the potential to prevent epidemics in contexts where relatively few individuals are vaccinated [5,14,15]. This characteristic makes transmissible vaccines an especially compelling option for blocking transmission and achieving herd immunity thresholds in populations likely to have low vaccination coverage; for example poultry pathogens in regions of sub-Saharan Africa with historically low vaccination coverage [16-18].

Field trials are crucial in the transmissible vaccine development pipeline, since they allow investigators to capture protections in animal populations at scale [13]. However, there is limited literature on randomized controlled trials (RCTs) [19], considered the gold standard in testing interventions [20], in evaluating transmissible vaccines. A methodology to capture the unique indirect protection of transmissible vaccines has not yet been proposed. To address this gap, we propose a two-stage randomized trial design for field trials of transmissible vaccines in animal populations, since in addition to the overall protective effect, it allows investigators to estimate with precision the significant indirect protection of these vaccines [1-3,21-25]. The estimation of these various protections of transmissible vaccines inform the debate of their more widespread use in animal populations.

While quantifying the protections provided by transmissible vaccines is of clear policy importance, the feasibility of such designs, specifically sample sizes required to adequately power such trials, remains an open question. Our article considers two plausible trial designs: trials that compare protection conferred by transmissible vaccines relative to traditional (i.e., non-transmissible) vaccines, as well as trials that estimate the overall protection of transmissible vaccines compared to pure control groups that do not receive any vaccination. Which of these designs is used in a given setting will depend on the available comparator(s) and the current standard, as well as economic and feasibility considerations, but can be informed by the statistical properties discussed here.

We make several methodological contributions towards the successful evaluation of transmissible vaccines using field trials. First, we extend the standard two-stage randomized trial design framework to allow for causal comparisons of two vaccine types: transmissible and non-transmissible vaccines. Second, we identify and characterize two novel statistical estimands that provide epidemiological insight into reductions in risk of infection for subpopulations unique to transmissible vaccines. Third, we use both simulation and analytic approximate sample size formulae to calculate and compare the required sample sizes needed to adequately power trials depending on vaccine characteristics (e.g., strength of transmissibility), trial characteristics (e.g., vaccination timing), and host ecology factors (e.g., contact structure of the animal host population). We show that, under many conditions, reasonable sample sizes for a single post-intervention sample can adequately estimate various protective effects. We also make these methods available for investigators to determine the required sample size for their setting. Finally, from this foundation, we discuss opportunities for further investigation into effectively evaluating the protections of transmissible vaccines in animal populations.

## Methods

The next three subsections describe the two-staged randomized trial design, estimands of interest, and estimators used, to evaluate the effects of a given transmissible vaccine. For sample size calculations, the following four subsections describe simulation and analytic sample size methods, grounded in an existing system. The simulation model is structured around transmission and contact structure parameters appropriate to the Newcastle disease virus transmission system in poultry, where the vaccine is a live attenuated form of the virus that is incidentally transmissible. The populations we study have no prior immunity, and the mean number of contacts for each animal is 15. The same methods for sample size estimation can be used for other pathogens or settings with appropriate changes to the parameters and model assumptions. In the Discussion, we consider how required sample sizes and trial designs may change across other study systems and pathogen and vaccine life-cycles. The trial design we propose is appropriate for field trials in the development pipeline of transmissible vaccines [13], which are proposed for geographically isolated areas such as islands or regions isolated by mountains to minimize the risk of vaccine transmission outside the study area.

### Trial design

**Fig 1:**
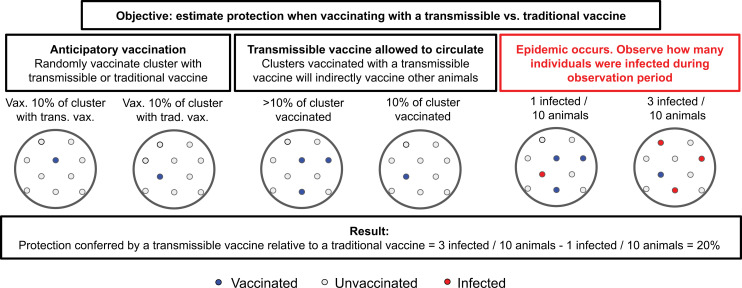
Simple example calculation of overall protection conferred by a transmissible vs. a traditional vaccine. First, clusters are vaccinated with either a transmissible or traditional vaccine. In the second stage, the transmissible is allowed to circulate for all animals. Finally, an epidemic occurs, and the number of infected animals of the cluster, among both initially vaccinated and initially unvaccinated animals, is counted. We assume there is no interference between clusters, i.e., The condition of vaccination or infection of an animal of one cluster affects either the condition of vaccination or probability of infection in another cluster. In this simple example 10% of each cluster is initially vaccinated for illustrative purposes, whereas in our Results 5% of each cluster is initially vaccinated.

**Fig 2:**
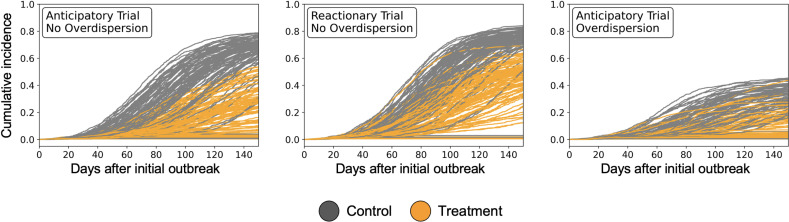
Differences in cumulative infection burden depend on whether the trial is anticipatory or reactionary, and if the contact structure is overdispersed. Left panel: anticipatory trial with no overdispersion in contact structure. Middle panel: reactionary trial, where vaccination starts once 0.5% of the population has been infected, with no overdispersion in contact structure. Right panel: anticipatory trial with overdispersion in contact stricture (k=1). Observation period is 150 days. Parameters set at: R_0,v_ = 1.1, R_0,w_ = 2, proportion initially recovered = 0, vaccine efficacy = 80%, initial vaccinated proportion, α = 5%.

We study trials with a two-stage randomized design, where in the first stage of the trial *N*_*T*_ clusters are randomized to either vaccinate α% = 5% of their cluster with a transmissible vaccine or a traditional vaccine, and in the second stage, animals are randomly assigned to be vaccinated with the vaccine assigned to the cluster in the first stage. An example schematic of the trial design is shown in Fig 1 [1]. We assume that vaccination coverage, α%, does not vary between treatment assignment strategies so that the estimands capture relevant quantities (see Section B.2.1 in S1 Text). The total number of recruited clusters, *N*_*T*_ *= N*_*0*_ *+ N*_*1*_ where *N*_*0*_ and *N*_*1*_ are the number of clusters in each arm; we set *N*_*0*_ = *N*_*1*_. We vary the transmissibility of the vaccine to be either weak (R_0,v_ = 0.9) or strong (R_0,v_ = 1.1). In the main results, for each R_0,v_, we model anticipatory trial designs, where the transmissible vaccine is allowed to circulate in the population before an outbreak of the wildtype pathogen. We also consider reactionary trial designs, where investigators react to an epidemic starting in a cluster and vaccinate susceptible animals when, on average, 0.5% of the population is actively infected (Fig 2) [5]. Additionally, we consider trials that compare protection from a transmissible vaccine to pure control groups that do not receive any vaccination.

150 days after first infection, 10% of animals in each cluster are randomly sampled and the cumulative number of infections in the sample is recorded. We choose a sampling fraction of 10% as a reasonable lower bound, since previous vaccination campaigns for poultry successfully sampled up to 50% of animals from farms at follow-up [26], and high sampling coverage for wildlife populations may be more difficult to achieve [12]. The ability to distinguish wildtype infection from vaccination is crucial, since statistical inference hinges on identifying decreases in wildtype infection burden. If infections are mild or subclinical, tools such as serological assays can be used to differentiate infected animals from vaccinated animals (DIVA) [27,28]. While the trial is not designed specifically to estimate the rate of occurrences of undesirable evolutionary outcomes, such as reversion to a pathogenic form, checks should be done during the trial itself to see whether these undesirable events have occurred. If animals that were solely seropositive for the vaccine, and not the wildtype pathogen, die at rates faster than we would expect naturally, this may be a sign that the vaccine has reverted to a pathogenic form.

### Estimation of causal protection

In the trials we consider there are several vaccine exposures: animals can either be initially vaccinated (also referred to as directly vaccinated) with either a traditional or transmissible vaccine, or initially unvaccinated; if the cluster has been treated with a transmissible vaccine, initially unvaccinated animals are either indirectly vaccinated through transmission of the vaccine or never vaccinated. To estimate the protections of a transmissible vaccine, we first introduce the estimand, $\Delta_{1}\left(\alpha \right)$ which defines the overall causal effect of protection that transmissible vaccines confer when *α*% of the population is initially vaccinated [1]. We define this estimand using the potential outcomes causal inference framework:


$$\Delta_{1}\left(\alpha \right)=\bar{Y}\left(\psi \left(\alpha \right)\right)-\bar{Y}\left(f\left(\alpha \right)\right)$$

where the estimand $\Delta_{1}\left(\alpha \right)$is an average of the difference in cluster/group potential outcomes of cluster i, ${\bar{Y}}_{i}\left(\psi \left(\alpha \right)\right)-{\bar{Y}}_{i}\left(f\left(\alpha \right)\right)$which are themselves differences in the averages of the individual potential outcomes for individual j of cluster i(further details in Section B.1.3 in S1 Text).

$\bar{Y}$ (.) is the population average potential outcome of infection under a given individual treatment assignment strategy.

$\psi \left(\alpha \right)$ is a treatment assignment strategy where *α*% of the population is initially vaccinated with a traditional vaccine.

$\Phi \left(\alpha \right)$is a treatment assignment strategy where *α*% of the population is initially vaccinated with a transmissible vaccine.

The estimator is:


$$\hat{\Delta_{1}\left(\alpha \right)}=\frac{1}{N_{0}}\sum_{u=1}^{N_{0}}Z_{\Delta_{1},\psi \left(\alpha \right),i=g\left(u\right)}\text{/}S_{\Delta_{1},\psi \left(\alpha \right),i=g\left(u\right)}-\frac{1}{N_{1}}\sum_{u=1}^{N_{1}}Z_{\Delta_{1},\Phi \left(\alpha \right),i=h\left(u\right)}\text{/}S_{\Delta_{1},\Phi \left(\alpha \right),i=h\left(u\right)}$$

where $S_{\Delta_{1},\psi \left(\alpha \right),i=g\left(u\right)}$ and $S_{\Delta_{1},\Phi \left(\alpha \right),i=h\left(u\right)}$ are the number of sampled animals from cluster *i* in the traditional arm ($\psi \left(\alpha \right)$) and transmissible arm ($\Phi \left(\alpha \right)$) of the trial, respectively.

$Z_{\Delta_{1},\psi \left(\alpha \right),i=g\left(u\right)}$ and $Z_{\Delta_{1},\Phi \left(\alpha \right),i=h\left(u\right)}$ are the number of sampled animals that were infected in the traditional arm ($\psi \left(\alpha \right)$) and transmissible arm ($\Phi \left(\alpha \right)$) of the trial, respectively.

g(u) and h(u) map the cluster index within each arm (from u = 1 to u = N_0_ or N_1_ for the traditional vaccine or transmissible vaccine arm, respectively) to the general cluster index, i.

For all causal estimands we assume that treatment assignment (i.e., initial vaccination) both on the cluster and individual levels is randomized, and that there is no interference between clusters, which is plausible for geographically isolated regions. We also assume perfect compliance, since animals would be directly vaccinated by investigators. Additionally, for all causal estimands, proper randomization of the treatment assignment, both on the cluster and individual levels, eliminates potential bias due to unmeasured confounders that have a relationship with both treatment assignment and outcome. Full details of all estimands, both causal and statistical (defined below), and estimators, including proofs of the unbiasedness, consistency, and asymptotic normality of the estimators, are provided in Appendix B in S1 Text.

For all estimators, we choose to estimate cluster average effects since we are interested in protections and risk differences that will depend on the cluster-level contact structure of a given cluster [29]. If cluster size, n, varies, the interpretation of our estimands should remain cluster-level averages to maintain interpretability, consistency, and validity.

We introduce two additional causal estimands: $\Delta_{2}\left(\alpha \right),$which defines the causal protection that transmissible vaccines confer to initially vaccinated individuals relative to initially vaccinated individuals using a traditional vaccine; and $\Delta_{3}\left(\alpha \right),$which defines the causal protection the transmissible vaccines confer to initially unvaccinated individuals relative to initially unvaccinated individuals using a traditional vaccine. Specifically, the estimands are:


$$\Delta_{2}\left(\alpha \right)=\bar{Y}\left(z=1;\psi \left(\alpha \right)\right)-\bar{Y}\left(z=2;\Phi \left(\alpha \right)\right)$$

$$\Delta_{3}\left(\alpha \right)=\bar{Y}\left(z=0;\psi \left(\alpha \right)\right)-\bar{Y}\left(z=0;\Phi \left(\alpha \right)\right)$$

where the estimand $\Delta_{2}\left(\alpha \right)$ is an average of the difference in cluster/group potential outcomes of cluster i, ${\bar{Y}}_{i}\left(z=1;\psi \left(\alpha \right)\right)-{\bar{Y}}_{i}\left(z=2;\Phi \left(\alpha \right)\right)$which are themselves differences in the averages of the individual potential outcomes for individual j of cluster i, ${\bar{Y}}_{ij}\left(z=1;\psi \left(\alpha \right)\right)-{\bar{Y}}_{ij}\left(z=2;\Phi \left(\alpha \right)\right)$

The estimand $\Delta_{3}\left(\alpha \right)$ is an average of the difference in cluster/group potential outcomes of cluster i, ${\bar{Y}}_{i}\left(z=0;\psi \left(\alpha \right)\right)-{\bar{Y}}_{i}\left(z=0;\Phi \left(\alpha \right)\right)$which are themselves differences in the averages of the individual potential outcomes for individual j of cluster i(further details in Section B.1.3 in S1 Text).

$\bar{Y}$ (.) is the population average potential outcome of infection under a given treatment assignment strategy for an individual of treatment assignment z.

z = 1 indicates an individual initially vaccinated with a traditional vaccine.

z = 2 indicates an individual initially vaccinated with a transmissible vaccine.

z = 0 indicates an individual initially unvaccinated.

$\psi \left(\alpha \right)$ and $\Phi \left(\alpha \right)$ are the same as for $\Delta_{1}\left(\alpha \right)$

Similar to the above overall causal effect, the population average potential outcome is an average of the group potential outcome which is itself an average of the individual potential outcome, defined as the infection outcome of individual j of initial vaccine exposure z of group i under the given treatment strategy. Note that because we do not model decreased infectiousness of vaccinated individuals, where vaccinated individuals infected with the wildtype pathogen have decreased subsequent transmission of the wildtype pathogen, the estimated indirect spillover effect *Δ*_3_(α) is due to the protective effect of contagion, where the prevention of a vaccinated individual from being infected with the wildtype pathogen prevents subsequent infection to their neighbors [30]. The estimators are:


$$\hat{\Delta_{2}\left(\alpha \right)}=\frac{1}{N_{0}}\sum_{u=1}^{N_{0}}Z_{\Delta_{2},\psi \left(\alpha \right),i=g\left(u\right)}\text{/}S_{\Delta_{2},\psi \left(\alpha \right),i=g\left(u\right)}-\frac{1}{N_{1}}\sum_{u=1}^{N_{1}}Z_{\Delta_{2},\Phi \left(\alpha \right),i=h\left(u\right)}\text{/}S_{\Delta_{2},\Phi \left(\alpha \right),i=h\left(u\right)}$$

$$\hat{\Delta_{3}\left(\alpha \right)}=\frac{1}{N_{0}}\sum_{u=1}^{N_{0}}Z_{\Delta_{3},\psi \left(\alpha \right),i=g\left(u\right)}\text{/}S_{\Delta_{3},\psi \left(\alpha \right),i=g\left(u\right)}-\frac{1}{N_{1}}\sum_{u=1}^{N_{1}}Z_{\Delta_{3},\Phi \left(\alpha \right),i=h\left(u\right)}\text{/}S_{\Delta_{3},\Phi \left(\alpha \right),i=h\left(u\right)}$$

where, for $\hat{\Delta_{2}\left(\alpha \right)}$$S_{\Delta_{2},\psi \left(\alpha \right),i=g\left(u\right)}$ and $S_{\Delta_{2},\Phi \left(\alpha \right),i=h\left(u\right)}$ are the number of sampled initially vaccinated animals in cluster *i* that are in the traditional arm ($\psi \left(\alpha \right)$) and transmissible arm ($\Phi \left(\alpha \right)$) of the trial, respectively.

$Z_{\Delta_{2},\psi \left(\alpha \right),i=g\left(u\right)}$ and $Z_{\Delta_{2},\Phi \left(\alpha \right),i=h\left(u\right)}$ are the number of sampled initially vaccinated animals that were infected in cluster *i* in the traditional arm ($\psi \left(\alpha \right)$) and transmissible arm ($\Phi \left(\alpha \right)$) of the trial, respectively.

Z and S expressions for $\hat{\Delta_{3}\left(\alpha \right)}$ are the same as for $\hat{\Delta_{2}\left(\alpha \right)}$except that they measure the proportion infected among initially unvaccinated animals (as specified by their subscript, *Δ*_3_).

### Estimation of differences in subpopulation risks of infection

Besides causal estimands, we introduce two statistical estimands to provide differences in the risk of infection for indirectly vaccinated animals—i.e., those animals who were not initially vaccinated and were subsequently vaccinated by the transmissible vaccine—and never vaccinated animals—i.e., those animals who were not initially vaccinated and did not get vaccinated by the transmissible vaccine—in clusters where the transmissible vaccine was implemented. We compare the risk of infections among these populations to the initially unvaccinated population among clusters where the transmissible vaccine was not implemented. We use θ to denote statistical estimands to differentiate them from Δ, which refers solely to causal estimands defined using potential outcomes (see Section B.3 in S1 Text). Similarly, we do not use the overline notation when defining these estimands since they are not potential outcomes.

Trials should aim to identify the causal effects of transmissible vaccines defined in “Estimation of causal protection.” However, if there are sufficient resources, these estimands provide further epidemiological information of the differences in risk of infection for subpopulations unique to transmissible vaccines. We caution that these estimands may not be directly interpretable as protection solely due to the individual-level status of indirect vaccination or never vaccination since there may be population-specific characteristics that can bias this interpretation. Since they use non-randomized groups in the transmissible vaccine arm, these estimates may be biased from the causal protective effect due to confounding of the transmission of vaccine and transmission of the pathogen. Rather than causal vaccine effects, these estimands represent true differences in the cluster-average risk of infection for subpopulations unique to the utilization of transmissible vaccines.

θ_1_(α) defines the difference in risk of infection between indirectly vaccinated animals in clusters that receive the transmissible vaccine, relative to unvaccinated animals in clusters that receive the traditional vaccine. We define the estimand as:


$$\theta_{1}\left(\alpha \right)=\frac{1}{N_{0}}\overset{N_{0}}{\underset{u=1}{\sum }}T_{\theta_{1},\psi \left(\alpha \right),i=g\left(u\right)}\text{/}K_{\theta_{1},\psi \left(\alpha \right),i=g\left(u\right)}-\frac{1}{N_{1}}\overset{N_{1}}{\underset{u=1}{\sum }}T_{\theta_{1},\Phi \left(\alpha \right),i=h\left(u\right)}\text{/}K_{\theta_{1},\Phi \left(\alpha \right),i=h\left(u\right)}$$

where $K_{\theta_{1},\psi \left(\alpha \right),i=g\left(u\right)}$ is the total number of initially unvaccinated animals in cluster i that are in the traditional vaccine arm ($\psi \left(\alpha \right)$) where α% of the cluster is initially vaccinated with a traditional vaccine and $T_{\theta_{1},\psi \left(\alpha \right),i=g\left(u\right)}$ is the number of those animals that were infected.

$K_{\theta_{1},\Phi \left(\alpha \right),i=h\left(u\right)}$ is the total number of indirectly vaccinated animals in the transmissible vaccine arm ($\Phi \left(\alpha \right)$) where α% of the cluster is initially vaccinated with a transmissible vaccine and $T_{\theta_{1},\Phi \left(\alpha \right),i=h\left(u\right)}$ is the number of those animals that were infected.

The functions g(u) and h(u) map the cluster index within each arm (from u = 1 to u = N_0_ or N_1_ for the traditional vaccine or transmissible vaccine arm, respectively) to the general cluster index, i. In other words, we take the difference in average cluster-level risks of infections among the two arms of the trial. The estimator is:


$$\hat{\theta_{1}\left(\alpha \right)}=\frac{1}{N_{0}}\sum_{u=1}^{N_{0}}Z_{\theta_{1},\psi \left(\alpha \right),i=g\left(u\right)}\text{/}S_{\theta_{1},\psi \left(\alpha \right),i=g\left(u\right)}-\frac{1}{N_{1}}\sum_{u=1}^{N_{1}}Z_{\theta_{1},\Phi \left(\alpha \right),i=h\left(u\right)}\text{/}S_{\theta_{1},\Phi \left(\alpha \right),i=h\left(u\right)}$$

where $S_{\theta_{1},\psi \left(\alpha \right),i=g\left(u\right)}$ is the number of sampled initially unvaccinated animals in cluster *i* that are in the traditional arm ($\psi \left(\alpha \right)$) and $Z_{\theta_{1},\psi \left(\alpha \right),i=g\left(u\right)}$ are the number of those sampled animals that were infected.

$S_{\theta_{1},\Phi \left(\alpha \right),i=h\left(u\right)}$ is the number of sampled indirectly vaccinated animals in the transmissible arm ($\Phi \left(\alpha \right)$) and $Z_{\theta_{1},\Phi \left(\alpha \right),i=h\left(u\right)}$ are the number of those sampled animals that were infected.

θ_2_(α) defines the difference in risk of infection between never vaccinated animals of clusters treated with the transmissible vaccine vs. initially unvaccinated animals of clusters treated with the traditional vaccine. We define the estimand as:


$$\theta_{2}\left(\alpha \right)=\frac{1}{N_{0}}\overset{N_{0}}{\underset{u=1}{\sum }}T_{\theta_{2},\psi \left(\alpha \right),i=g\left(u\right)}\text{/}K_{\theta_{2},\psi \left(\alpha \right),i=g\left(u\right)}-\frac{1}{N_{1}}\overset{N_{1}}{\underset{u=1}{\sum }}T_{\theta_{2},\Phi \left(\alpha \right),i=h\left(u\right)}\text{/}K_{\theta_{2},\Phi \left(\alpha \right),i=h\left(u\right)}$$

where $K_{\theta_{2},\psi \left(\alpha \right),i=g\left(u\right)}$ is the total number of initially unvaccinated animals in cluster i of the traditional arm ($\psi \left(\alpha \right)$) where α% of the cluster is initially vaccinated with a traditional vaccine and $T_{\theta_{2},\psi \left(\alpha \right),i=g\left(u\right)}$ is the number of those animals that were infected.

$K_{\theta_{2},\Phi \left(\alpha \right),i=h\left(u\right)}$ is the total number of never vaccinated animals (neither directly nor indirectly vaccinated) in cluster i of the transmissible arm ($\Phi \left(\alpha \right))$ where α% of the cluster is initially vaccinated with a transmissible vaccine and $T_{\theta_{2},\Phi \left(\alpha \right),i=h\left(u\right)}$ is the number of those animals that were infected.

The functions g(u) and h(u) map the cluster index within each arm (from u = 1 to u = N_0_ or N_1_ for the traditional vaccine or transmissible vaccine arm, respectively) to the general cluster index i. The estimator is:


$$\hat{\theta_{2}\left(\alpha \right)}=\frac{1}{N_{0}}\sum_{u=1}^{N_{0}}Z_{\theta_{2},\psi \left(\alpha \right),i=g\left(u\right)}\text{/}S_{\theta_{2},\psi \left(\alpha \right),i=g\left(u\right)}-\frac{1}{N_{1}}\sum_{u=1}^{N_{1}}Z_{\theta_{2},\Phi \left(\alpha \right),i=h\left(u\right)}\text{/}S_{\theta_{2},\Phi \left(\alpha \right),i=h\left(u\right)}$$

where $S_{\theta_{2},\psi \left(\alpha \right),i=g\left(u\right)}$ and are the number of sampled initially unvaccinated animals in cluster *i* that are in the traditional arm ($\psi \left(\alpha \right)$) and $Z_{\theta_{2},\psi \left(\alpha \right),i=g\left(u\right)}$ are the number of those sampled animals that were infected.

$S_{\theta_{2},\Phi \left(\alpha \right),i=h\left(u\right)}$ is the number of sampled never vaccinated animals in the transmissible arm ($\Phi \left(\alpha \right)$) and $Z_{\theta_{2},\Phi \left(\alpha \right),i=h\left(u\right)}$ are the number of those sampled animals that were infected.

Because of the complexity of variance estimation for clusters with different contact structures, all hypothesis testing is conducted using randomization-based inference; variance estimation and confidence intervals are calculated using resampling-based inference.

### Simulation model: cluster assumptions

We study trials that are conducted for clusters of moderately large size; the number of animals in each simulated cluster is size n = 1000. We define a cluster as a contiguous region where animals are in possible contact with one another, such as farms with free-roaming poultry. We assume that animals within each cluster can potentially interact through a network contact structure, but not between clusters [31,32]; thus, there is interference within clusters but not between clusters. Although all animals are expected to have a mean number of contacts, we assume the exact number is drawn from a distribution, and thus there is heterogeneity in the number of contacts per animal. Animals that were once infected or vaccinated cannot become infected or vaccinated again. Picking cluster characteristics that will reveal the protection and risk differences of transmissible vaccines is important; for example, in our case, picking larger n = 1000 animals ensures a higher probability of imported infection via exposure to external sources of infections [33,34]. We assume no dependency between cluster contact structure and importation rate, but note that investigators should be aware that this may potentially affect the importation rate of infections: if animals have a high number of contacts within clusters these animals may also have higher contact outside of the cluster, increasing infection importation. In practice, if n varies across the trial there may be informative cluster sizes where treatment effects differ across clusters [29]; our estimators would average over these treatment effects across clusters since they estimate cluster-average effects; interpretation of the causal effects would need to change accordingly. Within each cluster we assume that there is no prior immunity to wildtype infection and vaccination, as has been assumed in previous transmissible vaccine models [5,12].

We model the contact structure of the clusters using a network, where animals are nodes of the network, and edges represent contact between animals. The degree of each animal is either Poisson distributed (mean degree = 15) or moderately overdispersed (mean degree = 15, overdispersion parameter k = 1) with no nodes of degree 0 (Fig 2) [35,36]. We choose a mean degree of 15 to approximately match the average number of poultry per farm which experience outbreaks of poultry pathogens such as Newcastle disease virus and avian influenza [27,37]. While a Poisson-distributed degree distribution may be appropriate to model certain ecological contexts, we additionally model a moderately overdispersed degree distribution (contact structure) due to the overdispersion that has been observed in the connectivity of hosts on a regional scale, e.g., within a trading network where Newcastle disease virus circulates [38]. We assume that both the wildtype pathogen and vaccine transmit on this same contact structure of the population.

For overdispersed contact structures, in order to create each cluster according to the desired degree distribution, we use a configuration model (CM) algorithm that does not allow singletons in the cluster, nor self-loops and multi-edges between nodes [39]. Because of our modifications, the effective degree distribution of the cluster nearly, but does not exactly, match the desired degree distribution. The number of connected components in each cluster is low; we have observed at most three connected components in a single cluster. Thus, we account for the possibility of disjoint groupings of animals within a single cluster, where there is no interference, which may occur in practice.

### Simulation model: wildtype and vaccine transmission assumptions

We model transmission through an animal population with a continuous-time, stochastic SEIR model (Fig A in S1 Text) [40]. Although our aim is to provide results that are applicable across a broad spectrum of livestock and wildlife pathogens, we initially model an acute infectious pathogen similar to poultry pathogens that infect chickens, and discuss generalization of these results below. In the model, an animal’s mean incubation period and mean infectious period are 5 days and 10 days respectively, both for the wildtype pathogen and vaccine, mirroring the average incubation and infectious period of Newcastle disease virus [41,42]. The wildtype infection and vaccine infection differ solely in their rate of transmission. The incubation period, infectious period, and time between onward transmission events are exponentially distributed [43]. The memoryless property of the exponential distribution allows us to interrupt the epidemic simulation to implement vaccination in reactionary trials without interfering with the epidemic progression. We assume R_0,w_, or the R_0_ of the wildtype strain, is 2 in order to model a highly transmissible wildtype strain, such as Newcastle disease virus [38,44]. Parameter values and references are detailed in Table A in S1 Text.

We assume that the vaccine confers either perfect immunity or “leaky” immunity, where the vaccine efficacy for vaccinated animals against wildtype infection is 80% (i.e., their risk of infection on each contact is reduced by 80%). We assume vaccinated animals cannot subsequently become reinfected by the vaccine. Additionally, animals infected with the wildtype cannot become reinfected by either the wildtype or vaccine. We do not model vaccine failure, where some proportion of vaccinated animals do not have any protection against infection (“all-or-nothing” vaccine effects). Similarly, we do not model vaccine transmission failure, where the vaccine systematically fails to transmit for some vaccinated animals, nor a decreased infectiousness of vaccinated individuals [30]. We assume both immunity from wildtype and vaccine infection last for the entire trial duration. In our simulations, there is also no superinfection, meaning that no animal can simultaneously be infected by both the vaccine and wildtype strain.

We seed the epidemic of each cluster with four initial infections. Both when vaccinating 5% of susceptible animals at intervention and when seeding the epidemics with wildtype infection, we select animals randomly to vaccinate and infect, respectively. We use the *EoN* python package to simulate the epidemic [45]. Code used for simulations is provided at http://www.github.com/jsheen/netVax.

### Required sample sizes, N_T_* through simulation

For each estimand in a given scenario we report the required sample size, N_T_*, which we define as the minimum number of total clusters across both arms of the trial needed to achieve at least 80% power to detect a statistically significantly lower infection burden (p-value ≤ 0.05) in the transmissible vaccine treatment arm compared to the second arm of the trial, which is either a traditional vaccine treatment or pure control with no vaccination (one-sided test). The estimands across scenarios are for the overall causal effect $\Delta_{1}\left(\alpha \right)$ and spillover causal effect $\Delta_{3}\left(\alpha \right)$, as well as the differences in risk for indirectly vaccinated animals and never vaccinated animals, which we refer to as the statistical estimands (see “Estimation of causal protection” and “Estimation of differences in subpopulation risks of infection”). The total vaccine causal effect on the vaccinated $\Delta_{2}\left(\alpha \right)$ could be measured here, but is more relevant when vaccine efficacy varies between arms of the trial; we leave that exploration to future work. For a given number of clusters, the power of the trial is the proportion of 1000 simulated trials with p-value ≤ 0.05 under the alternative hypothesis of a true effect. To obtain the p-value of each simulated trial for hypothesis testing, we conduct a randomization (permutation) test. We first create a null distribution by performing 1000 random permutations of the original labels of clusters to each of the two arms of the trials, in each permutation recalculating the targeted estimate of protection or difference in risk of infection. When there are 12 or fewer clusters, all possible permutations are used instead of randomly choosing 1000. The p-value is:


$$\text{p}-\text{value}=1-{\text{p}}_{\text{n}}$$

where p_n_ is the proportion of the estimates in this null distribution that are less than or equal to the original estimate.

To identify N_T_*, we start with eight clusters as the lower bound on the number of clusters across both arms of the trial and increase by two clusters, one for each arm of the trial, until 80% power is achieved. A minimum of eight clusters is used since this is the minimum number of clusters needed for the permutation test to be able to yield a p-value under 0.05. Note that because of the coarse scale of the evaluation of the design (e.g., adding relatively large clusters), the power obtained can be noticeably above 80%. To ensure statistical validity, we also provide the Type I error rate of N_T_*, i.e., the false positive rate, by calculating the power of the trial if the vaccine has no effect across 3000 trial simulations.

For each N_T_* for each estimand of a given scenario, we also provide the mean estimate of the protective effect, or risk difference, and mean width of the bootstrapped 95% confidence interval for each estimate. We choose a bootstrapped confidence interval to estimate variance since existing analytical variance estimators may not be appropriate in our study setting, for example due to the assumption of stratified interference [1]. To obtain the 95% confidence intervals of each estimate, we create 1000 bootstrap samples and find the 2.5 and 97.5 percentiles of the samples. To create each bootstrap sample, we first draw with replacement the clusters of each arm of the trial in the original sample [46,47]. Next, we draw with replacement the same number of sampled animals, s, from each resampled cluster, which may be a conservative estimate of the variance [48-49]. Alternatively, investigators may also include all originally sampled animals from each resampled cluster, among other resampling approaches [46].

Clusters where less than three sampled animals (i.e., less than 3% of the sample) were observed to have an infection are excluded in our analysis. For cluster randomized trials and infectious disease outbreak studies more generally, bias or reduced efficiency can arise from including communities with few or no events [50,51]. Observing at least three infected animals in our sample indicates transmission in the population, ensuring exposure to the wildtype pathogen, and improving the efficiency of estimation and power of the hypothesis test, especially for overdispersed populations. If the vaccine has no effect, the exclusion should not introduce any bias. When the vaccine is effective enough to cause less than 3% of the population to be infected on average, the exclusion may create a downward bias by excluding clusters where the vaccination successfully prevented almost all transmission. Thus, the exclusion can potentially raise N_T_* in those settings, since treated clusters with little to no infection will not be used for analysis. However, because infection rates for highly transmissible pathogens such as Newcastle disease virus are high [17], and because we model settings where a moderately small portion of the population is vaccinated (5%), this scenario is unlikely, and we specifically study these scenarios to avoid bias. With a larger initially vaccinated population or even more transmissible vaccine, where the vaccinated proportion may reach larger proportions of the population, the bias and efficiency loss may be more pronounced since there may be more clusters with little to no infection (noting that this will yield a conservative estimate of N_T_*). Our reported sample sizes account for the potential exclusion: for a given N_T_* across all trial simulations at least 80% are statistically significant, even if for any one trial simulation clusters may be excluded before analysis. When calculating the risk difference for indirectly vaccinated animals (θ_1_(α)), we do not use this constraint, since the effect necessarily relies on vaccinated animals who have little to no risk of infection.

### Approximate formula for the required sample size, N_T_*

In addition to the simulation study results, which are based on the power of a permutation test to detect a significant risk difference of infection between arms of the trial, we also provide an analytical solution for the approximate required sample size, N_T_*, to adequately power trials that use a Welch’s t-test on the risk differences of infection between a transmissible vaccine and traditional vaccine. When the traditional vaccine confers no protection, this comparison is equivalent to comparing the protection or infection risk of transmissible vaccines to control groups that do not receive any vaccine. This approximate sample size formula demonstrates how N_T_* depends on both ecological and study design factors such as the R_0_ of the vaccine, R_0_ of the wildtype pathogen, the cluster size, n, number of animals sampled from each cluster, s, the initial recovered population, initial vaccinated population, vaccine efficacy, and the between-cluster variance in final size proportions. Although the approximate formula is useful for understanding sample size relationships, in practice, because the variances of the estimators we calculate for the approximate formula are lower bounds based on distributional and epidemic assumptions (see Appendix C in S1 Text) [52], the bootstrapped confidence intervals defined in the simulation study methods above are a more appropriate estimate of variance. All methods to derive the formula are provided in S2 Appendix C in S1 Text. An R Shiny app that shows the changes in N_T_* according to this formula is available at https://j-k-s.shinyapps.io/shinyTrans/.

## Results

The required sample sizes across both arms of the trial, N_T_*, to achieve 80% power for anticipatory trials in non-overdispersed settings when comparing the overall and spillover causal effects of a transmissible vaccine with R_0,v_ = 0.9 or 1.1 vs. a non-transmissible traditional vaccine when the vaccine efficacy is either 80% or 100% are broadly logistically feasible (Table 1) [26,53,54]. If there are sufficient resources, the risk differences for subpopulations unique to transmissible vaccines are also logistically feasible to test (Table 1). The minimum number of total animals that need to be sampled is 800 animals from 8 clusters, and the maximum number of total animals that would need to be sampled is 1800 animals from 18 clusters when the vaccine has 80% efficacy and is weakly transmissible (Table 1). The Type I error across all scenarios is ≤ 5%.

**Table 1:** Estimated protection or risk difference from and required sample sizes, N_T_*, for trials comparing a transmissible vaccine to a traditional vaccine. Prior to the outbreak, α%=5% of each cluster is vaccinated with either a transmissible or traditional, non-transmissible vaccine. The contact structure is Poisson distributed and trials are anticipatory. The estimand of interest is noted in the first column: Δ_1_(5) refers to the overall protection, Δ_3_(5) refers to the spillover protection, θ_1_(5) refers to the difference in risk of infection for indirectly vaccinated animals in the transmissible case compared to those unvaccinated in the traditional case, and θ_2_(5) refers to the difference in risk of infection for never vaccinated animals in the transmissible case. Vax. Eff. is the true direct efficacy of the vaccine to decrease susceptibility of vaccinated animals. R_0,v_ refers to the R_0_ of the vaccine. Estimate protection or risk difference is the mean estimate of the noted estimand comparing transmissible vaccines to traditional vaccines. <.> indicates the mean 95% confidence interval width. Req. sample size from simulation, N_T_*, is the number of clusters and number of animals required to estimate the effect in order to achieve at least 80% power. Power is the percentage of simulations with a p-value ≤ 5% across 1000 trial simulations. Type I error is the percentage of simulations with a p-value ≤ 5% across 3000 trial simulations for this sample size with a traditional vaccine.

	Vax. Eff.	R_0,v_	Estimate protection or risk difference: mean <CI>	Req. sample size from simulation, N_T_*	Power	Type I Error
Δ_1_(5)	0.8	0.9	25% <25%>	10 clus., 1000 indiv.	81%	4%
		1.1	41% <26%>	10 clus., 1000 indiv.	95%	4%
	1	0.9	32% <26%>	10 clus., 1000 indiv.	91%	4%
		1.1	49% <24%>	10 clus., 1000 indiv.	92%	4%
Δ_3_(5)	0.8	0.9	26% <26%>	10 clus., 1000 indiv.	83%	4%
		1.1	42% <27%>	10 clus., 1000 indiv.	95%	4%
	1	0.9	33% <27%>	10 clus., 1000 indiv.	91%	4%
		1.1	52% <25%>	10 clus., 1000 indiv.	94%	5%
θ_1_(5)	0.8	0.9	58% <30%>	8 clus., 800 indiv.	80%	4%
		1.1	64% <23%>	8 clus., 800 indiv.	80%	4%
	1	0.9	69% <19%>	8 clus., 800 indiv.	91%	4%
		1.1	70% <18%>	8 clus., 800 indiv.	90%	4%
θ_2_(5)	0.8	0.9	19% <21%>	18 clus., 1800 indiv.	81%	5%
		1.1	35% <30%>	10 clus., 1000 indiv.	88%	4%
	1	0.9	26% <27%>	12 clus., 1200 indiv.	86%	4%
		1.1	45% <29%>	10 clus., 1000 indiv.	91%	4%

N_T_* depends on the estimand of interest. N_T_* for the overall and spillover causal effects were found to be the same for anticipatory trials in non-overdispersed settings (Table 1). Additional estimation of the difference in risk of infection for indirectly vaccinated animals (θ_1_(5)) requires the smallest sample size (approximately 800 animals from 8 clusters), while estimating the difference in risk of infection for never vaccinated animals (θ_2_(5)) requires the largest (approximately 10–18 clusters sampling 1000–1800 animals total).

Mean effect estimates increase as the vaccine transmissibility and efficacy increase. For example, the overall causal effect increases from 25% to 41% with increased transmissibility from 0.9 to 1.1 when the vaccine efficacy is leaky and increases from 32% to 49% when the vaccine is perfectly effective instead of 80% effective. These increases in vaccine transmissibility and vaccine efficacy can reduce N_T_*, which may be crucial in resource-limited areas. For most scenarios the mean 95% bootstrapped confidence interval width was less than or equal to the mean effect estimate.

When the contact structure is overdispersed (Table 2), or when trial designs are reactionary (Table 3), N_T_* for the causal effects, as well as estimates of risk differences for subpopulations unique to transmissible vaccines, can increase due to more clusters with little to no infections in the former, and increased competition of the vaccine with the wildtype pathogen for susceptible animals (and, thus, less opportunity for indirect protection) in the latter. For overdispersed populations, even for strongly transmissible vaccines, N_T_* can increase from 10 clusters sampling 1000 animals total to 22 clusters sampling 2200 animals total. Similarly, when the vaccine is weakly transmissible and vaccine efficacy is imperfect, if the trial design is reactionary rather than anticipatory, N_T_* for the overall causal effect increases from 10 clusters sampling 1000 animals to 18 clusters sampling 1800 animals total. But although sample sizes increase in these situations, they remain feasible as they are no greater than twice the maximum number we would expect to sample across all three estimands in our best case scenario of anticipatory trials in environments with non-overdispersed contacts (Table 1).

**Table 2:** Estimated overall protection or risk difference from and required sample sizes, N_T_*, for trials comparing a transmissible vaccine to a traditional vaccine when clusters have overdispersed contact structures. Prior to the outbreak, α%=5% of each cluster is vaccinated with either a transmissible or traditional, non-transmissible vaccine. The contact structure is moderately overdispersed (k=1) and trials are anticipatory. The estimand of interest is noted in the first column: Δ_1_(5) refers to the overall protection, Δ_3_(5) refers to the spillover protection, θ_1_(5) refers to the difference in risk of infection for indirectly vaccinated animals in the transmissible case compared to those unvaccinated in the traditional case, and θ_2_(5) refers to the difference in risk of infection for never vaccinated animals in the transmissible case. Vax. Eff. is the true direct efficacy of the vaccine to decrease susceptibility of vaccinated animals. R_0,v_ refers to the R_0_ of the vaccine. Estimate Protection or risk difference is the mean estimate of the noted estimand comparing transmissible vaccines to traditional vaccines. <.> indicates the mean 95% confidence interval width. Req. sample size from simulation, N_T_* is the number of clusters and number of animals required to estimate the effect in order to achieve at least 80% power. Power is the percentage of simulations with a p-value ≤ 5% across 1000 trial simulations. Type I error is the percentage of simulations with a p-value ≤ 5% across 3000 trial simulations for this sample size with a traditional vaccine.

	Vax. Eff.	R_0,v_	Estimate protection or risk difference: mean <CI>	Req. sample size from simulation, N_T_*	Power	Type I Error
Δ_1_(5)	0.8	1.1	19% <18%>	22 clus., 2200 indiv.	80%	5%
Δ_3_(5)	0.8	1.1	20% <19%>	22 clus., 2200 indiv.	84%	5%
θ_1_(5)	0.8	1.1	24% <24%>	14 clus., 1400 indiv.	84%	5%
θ_2_(5)	0.8	1.1	19% <19%>	24 clus., 2400 indiv.	83%	5%

**Table 3:** Estimated overall protection from and required sample sizes, N_T_* for reactionary trials comparing a transmissible vaccine to a traditional vaccine. When 0.5% of the cluster is infected, α%=5% of each cluster is vaccinated with either a transmissible or traditional, non-transmissible vaccine. The contact structure is Poisson distributed. Δ_1_(5) is the overall protection. Vax. Eff. is the true direct efficacy of the vaccine to decrease susceptibility of vaccinated animals. R_0,v_ refers to the R_0_ of the vaccine. Estimate protection is the mean estimate of the overall protection conferred by transmissible vaccines compared to traditional vaccines. <.> indicates the mean 95% confidence interval width. Req. sample size from simulation, N_T_*, is the number of clusters and number of animals required to estimate the effect in order to achieve at least 80% power. Power is the percentage of simulations with a p-value ≤ 5% across 1000 trial simulations. Type I error is the percentage of simulations with a p-value ≤ 5% across 3000 trial simulations for this sample size with a traditional vaccine.

	Vax. Eff.	R_0,v_	Estimate protection: mean <CI>	Req. sample size from simulation, N_T_*	Power	Type I Error
Δ_1_(5)	0.8	0.9	14% <16%>	18 clus., 1800 indiv.	82%	5%
		1.1	23% <23%>	10 clus., 1000 indiv.	84%	4%

N_T_* of approximately 10 clusters sampling 1000 animals total for the overall causal effect do not change for trials that compare the transmissible vaccine to pure control groups that do not receive any vaccine (Table 4). Both the power and the estimate of protection increase in this scenario, and the greater power in these cases may indicate that for other parameter settings N_T_* would be lower.

**Table 4:** Estimated overall protection from and required sample sizes, N_T_*, for anticipatory trials comparing a transmissible vaccine to a pure control group with no vaccination. Clusters are either vaccinated at a rate of α%=5% prior to the outbreak with a transmissible vaccine or do not receive any vaccination. The contact structure is Poisson distributed and trials are anticipatory. Δ_1_(5) is the overall protection. Vax. Eff. is the true direct efficacy of the vaccine to decrease susceptibility of vaccinated animals. R_0,v_ refers to the R_0_ of the vaccine. Estimate protection is the mean estimate of the overall protection conferred by transmissible vaccines compared to traditional vaccines. <.> indicates the mean 95% confidence interval width. Req. sample size from simulation, N_T_*, is the number of clusters and number of animals required to estimate the effect in order to achieve at least 80% power. Power is the percentage of simulations with a p-value ≤ 5% across 1000 trial simulations. Type I error is the percentage of simulations with a p-value ≤ 5% across 3000 trial simulations for this sample size with a traditional vaccine.

	Vax. Eff.	R_0,v_	Estimate protection: mean <CI>	Req. sample size from simulation, N_T_*	Power	Type I Error
Δ_1_(5)	0.8	0.9	30% <25%>	10 clus., 1000 indiv.	95%	4%
		1.1	47% <26%>	10 clus., 1000 indiv.	97%	4%

### Approximate sample size formula results

**Fig 3:**
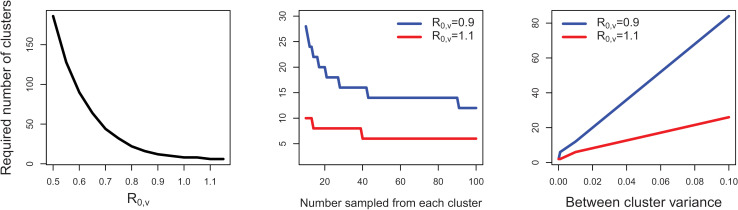
Required sample sizes, N_T_*, for overall protection estimand (Δ_1_(5)) will depend on the transmissible vaccine’s protective strength, as well as on the trial design. Our approximate formula reveals that as the R_0_ of the vaccine increases (left panel) or number sampled in each cluster, s, increases (middle panel), N_T_* for trials that measure the overall protection that transmissible vaccines confer relative to traditional vaccines will decrease. N_T_* will increase as the between-cluster variance in the final size proportion increases. All other parameters are set at R_0,v_ = 1.1, number sampled from each cluster, s = 100, between cluster variance = 0.01, R_0,w_ = 2, proportion initially recovered = 0, vaccine efficacy = 80%, initial vaccinated proportion, α = 5%.

Fig 3 shows the relationships between N_T_* estimated by an approximate analytic formula for trials of the overall causal effect of transmissible vaccines (Δ_1_(5)), with factors unique to transmissible vaccines, as well as with the trial design. As the R_0,v_ of the vaccine increases or the between-cluster variance decreases, N_T_* decreases. Similarly, we find that as the R_0_ of the wildtype pathogen increases, N_T_* first decreases, since larger exposure of the pathogen will highlight differences in protection conferred by the two vaccine types, then eventually increases as differences in the final proportion of infected individuals between the two vaccine types decrease (Fig C in S1 Text). Thus, although exposure to the pathogen is necessary to detect a protective effect, as the R_0_ of the wildtype pathogen increases, it is harder to observe differences in the proportions of infected individuals. Decreasing the vaccine efficacy will also increase sample sizes (Fig C in S1 Text). Sampling more animals from clusters can increase the power to detect the protection of these transmissible vaccines, and sampling less from more clusters may have higher power than sampling more from fewer clusters (middle panel Fig 3).

## Discussion

The development and utilization of transmissible vaccines for protection of both wildlife and domestic animal populations against pathogens remains a contentious issue [9]. Identifying the protective effects conferred by transmissible vaccines relative to traditional vaccines in real-world epidemiological conditions is necessary for the successful development of transmissible vaccines [13,15,20]. Towards this aim, we propose a field trial design that utilizes causal inference methods to capture the overall, spillover, and vaccine protective effects of transmissible vaccines. We also provide estimates of differences in the risks of infection for subpopulations unique to transmissible vaccines: animals indirectly vaccinated with the transmissible vaccine, and never vaccinated animals. Trials should be powered to identify causal effects, but with sufficient resources, differences in risk of infection for subpopulations unique to transmissible vaccines can also be estimated. We prove the unbiasedness,consistency, and asymptotic normality for estimators of both causal and statistical effects and perform a simulation study of several of the estimators to test the feasibility of this trial design in realistic epidemic scenarios [1]. In our simulations we vary the timing of intervention, transmissibility of the vaccine, efficacy of the vaccine, contact structure of the population.

We find that the sample sizes needed to adequately power our proposed anticipatory trial design to capture overall and spillover effects are feasible in groups of animals with non-overdispersed contact structures, since approximately 10 clusters sampling 1000 animals total are needed to adequately power the trial. Although trials should primarily aim to estimate the overall and spillover causal effects of the transmissible vaccine, such trials would be adequately powered to estimate the difference in risk of infection for indirectly vaccinated animals (required sample size, N_T_* = 8 clusters sampling 800 animals total) and if the sample size increases to 10–18 clusters sampling 1000-1800 animals total, depending on the vaccine efficacy and R_0_ of the transmissible vaccine, the trial would capture the difference in risk of infection for never vaccinated animals. When the trial compares transmissible vaccines to pure control groups that receive no vaccination, N_T_*e for the overall causal effect remains at 10 clusters sampling 1000 animals total but with higher than the nominal power. If investigators use the maximum N_T_* across all four estimands (overall causal effect, spillover causal effect, difference in risk for indirectly vaccinated animals, and difference in risk for never vaccinated animals) for a given scenario, all four estimands can be estimated, although this ignores multiple testing effects. Through an approximate sample size formula we derive, we also find that N_T_* needed to power these trials will decrease with increases in the strength of the transmissible vaccine, R_0,v_, number sampled from each cluster, s, and vaccine efficacy. These sample sizes will also decrease with decreases in the between cluster variance of the proportion infected and R_0_ of the wildtype pathogen. These results can be used to quickly estimate N_T_* for other settings, before confirmation using a full simulation model.

Estimates of these effects may be especially sensitive to cluster-level factors such as heterogeneity in contact structure. Despite this sensitivity, we find that generally, N_T_* needed to power trials in order to capture the protective effects are achievable, even for weakly transmissible vaccines [14]. Many real-world populations for which transmissible vaccines have been proposed are expected to contain heterogeneities in contact rates, yet measurement of protection is still feasible, at most 10 cluster sampling 1000 animals total for the overall causal effect [55]. If vaccines compete with the wildtype pathogen for susceptible animals, as they do in reactionary trials, protection could decrease, increasing N_T_* to 18 clusters sampling 1800 animals total. Similarly, in environments with overdispersed contact structures, N_T_* may increase to sampling 22 clusters sampling 2200 animals total. The trial design remains feasible even for these more challenging settings, as previous campaigns and field trials for poultry vaccines have been able to vaccinate hundreds, thousands, and in one case over hundreds of thousands of poultry at the village level within a year [26,53,54]. Given this historical precedent of large scale campaigns of poultry vaccination, the sample sizes we propose, from the hundreds to thousands, are comparable and thus logistically feasible.

Our results show that anticipatory trials may be a useful tool to decrease N_T_* to power the trial, but that sample size increases may be required to buffer against unknown parameters, such as the exact contact structure of the cluster and transmissibility of the vaccine, when conducting the trial. In such cases, investigators could choose the maximum sample size across all possible scenarios to minimize the risk of conducting an underpowered trial. To estimate the overall protective effect, this amounts to 22 clusters sampling 2200 animals total, which more than doubles the number of animals from the lower bound of 10 clusters sampling 1000 animals total. The most practical trial design will depend on these three ecological factors. For example, in cases where the R_0_ of the wildtype is high, such as for poultry pathogens (thus raising N_T_*, as shown in the analytical sample size results), investigators may prefer to estimate the effects of strongly transmissible vaccines, rather than weakly transmissible vaccines (all else being equal), in order to decrease N_T_*.

Although evolution within the vaccine strain might first be identified in laboratory studies, with monitoring of health outcomes across individuals the field trial designs we derive allow us to observe if unintended evolution occurs at scale [13]. Further, the protective efficacy estimates from these trials can be used to quantify the protective benefits of these vaccines in order to weigh them against potential unintended evolution. For example, with the trial design and setting we propose, strongly transmissible vaccines with 80% efficacy are expected to reduce the infection burden of the cluster by 41% on average compared to traditional vaccines. The 41% overall protection beyond that of the traditional vaccine can then be weighed against the potential for unintended evolution. If a second trial can be done comparing a weakly transmissible vaccine to a traditional vaccine, these results can also be used to weigh against the evolutionary risk of using a vaccine with higher transmissibility. For example, weakly transmissible vaccines are expected to reduce infection burden by 25% compared to a traditional vaccine, and this reduction can be compared to the 41% reduction of strongly transmissible vaccines. Future work that makes further assumptions of rates of reversion to wildtype could address which of the proposed estimands is most relevant for assessing unintended evolution and what sample size is required to estimate it. These characterizations would lend further support (if there was no observed undesirable evolution) or strong discouragement for a proposed transmissible vaccine (if there was observed undesirable evolution).

The trial design we propose is most suitable for populations where the wildtype pathogen is newly introduced in the population, sparking an epidemic, rather than circulating endemically. For example, since the wildtype rabies virus circulates endemically in these populations, standard epidemiological theory would predict that the vaccine strain will not circulate greatly in the population if it has a lower R_0_ than the wildtype, which is usually the case [56-58]. When pathogens are more episodic, there are periods without circulating pathogen, and thus the vaccine has time to spread further in the population without competing with the wildtype pathogen for susceptible animals. Thus, transmissible vaccines against poultry pathogens which create seasonal outbreaks may be an appropriate candidate for such trials. Future work may study how characteristics of endemicity, such as prior immunity, can affect the sample sizes we provide.

The models used here can also be extended to account for setting-specific effects, such as the distribution of immunity within explicit subpopulations and less than perfect protectiveness of prior immunity. Modeling explicit sub-clusters, where there is greater within-cluster connectivity and less between-cluster connectivity may also change our results, since transmissible vaccines may be more easily transmitted within sub-clusters, and less easily transmitted between clusters. Since contact structures for poultry networks as well as bat colonies have been constructed [39,59] ]these more specific dependencies can potentially be incorporated in future work. Informative cluster size may also increase N_T_* if the rate of imported infections decreases, and thus avoiding recruitment of smaller clusters may be needed to mitigate this risk. Characteristics of leaky vaccines may affect N_T_*: while vaccinated animals may still get infected if vaccine efficacy is imperfect, the vaccine may still reduce infection severity, decreasing the time of the infectiousness period and probability of forward transmission. This may further reduce infection burden and N_T_* if transmissible vaccines spread far in a population. Finally, the approximate sample size formula assumes normality of the effect and null distributions, and deterministic solutions for the final size of outbreaks, in contrast to the simulation study results. Thus it may be difficult to directly compare these results to N_T_* from the simulation study. Accounting for these differences may allow more direct comparison between the analytical solution and simulation study results. Both the simulation results as well as analytical results we provide can easily be extended to test other epidemiological settings and design features; the code we use and instructions for how to implement it is freely available in the Github repository we provide. For example, in this study we obtain the required sample size to adequately power the trial, but the code we provide can also be used to identify the required sample size to reduce the confidence interval uncertainty to a specific width.

The statistical estimands provide epidemiological information for indirectly vaccinated animals, θ_1_(5), and never vaccinated animals, θ_2_(5), in clusters where the transmissible vaccine is implemented and are potentially useful for modeling and surveillance. We leave to future work detailed characterization of the interpretation of these estimands and their relationship with causal protective effects for these groups. As these estimands do not lend themselves to the exchangeability assumption on the individual level since indirect vaccination is not randomly applied, and thus there is potential confounding, observational study methods may be useful to estimate causal effects. For example, if the degree of animals is known, then a matching could be done on degree to account for the potential confounder of higher degree animals being more likely to be indirectly vaccinated as well as infected with the wildtype pathogen. Identification of these causal estimands within an exposure-mediation interaction framework, where the mediator is whether or not an initially unvaccinated animal has been indirectly vaccinated, may also be successful [60]. Accounting for explicit network structures into these estimands is also warranted, given that they will certainly depend on the underlying network contact structure.

In this paper, we have laid the groundwork for the design of field trials to evaluate transmissible vaccines. Future studies that aim to identify ways to relax assumptions of this study could help further frame and specify trial designs needed for the potential introduction of this promising tool to lower the infection burden for many wildlife and livestock animal populations.

## Supplementary information

S1 Text Appendix A: Additional Figures and Tables. Appendix B: Estimands and Estimators Appendix C: Approximate sample size formula Fig A: SEIR model with vaccination. Fig B: Direct and indirect protection of a transmissible vaccine broken down by individual. Fig C: Required sample sizes, N_T_*, depend on R_0_ of the wildtype pathogen as well as the vaccine efficacy. Table A: Epidemiological parameter values. Table B: Estimated overall protection from and required sample sizes, N_T_*, for trials comparing a transmissible vaccine to a traditional vaccine when cluster size n = 500.
(PDF)
