# Correction: Design of field trials for the evaluation of transmissible vaccines in animal populations

**DOI:** 10.1371/journal.pcbi.1013530

**Published:** 2025-09-26

**Authors:** The PLOS Computational Biology Staff

In the Estimation of causal protection subsection of the Methods, there is an error in the first equation in the second term and the following text below the equation. The f(alpha) should read as phi(alpha). Please view the complete, correct equations here:


$$\Delta_{\text{1}}(\alpha )=\overset{―}{Y}\left(\psi (\alpha )\right)-\overset{―}{Y}\left(\phi (\alpha )\right)$$

$${\overset{―}{Y}}_{i}\left(\psi (\alpha )\right)-{\overset{―}{Y}}_{i}\left(\phi (\alpha )\right)$$

The publisher apologizes for the errors.
