# Supplementary material for: Design of field trials for the evaluation of transmissible vaccines in animal populations
Source: PLoS Comput Biol. 2025 Feb 3;21(2):e1012779. doi: 10.1371/journal.pcbi.1012779 (PMC11790233; doi:10.1371/journal.pcbi.1012779)
Supplement: S1 Text — Appendix A: Additional Figures and Tables. Appendix B: Estimands and Estimators Appendix C: Approximate sample size formula Fig A: SEIR model with vaccination. Fig B: Direct and indirect protection of a transmissible vaccine broken down by individual. Fig C: Required sample sizes, NT*, depend on R0 of the wildtype pathogen as well as the vaccine efficacy. Table A: Epidemiological parameter values. Table B: Estimated overall protection from and required sample sizes, NT*, for trials comparing a transmissible vaccine to a traditional vaccine when cluster size n = 500. (PDF) [file pcbi.1012779.s001.pdf]

Supporting Information for: "Design of field trials for the evaluation of transmissible vaccines in animal populations"

Justin K. Sheen[1*], Lee Kennedy-Shaffer[2,3], Michael Z. Levy[4], and C. Jessica E. Metcalf[1,5]

[1]Department of Ecology and Evolutionary Biology, Princeton University, Princeton, NJ, USA
[2]Department of Mathematics and Statistics, Vassar College, Poughkeepsie, NY, USA
[3]Department of Biostatistics, Yale School of Public Health, New Haven, CT, USA
[4]Department of Biostatistics, Epidemiology and Informatics, University of Pennsylvania Perelman School of Medicine, Philadelphia, PA, USA
[5]School of Public and International Affairs, Princeton University, Princeton, NJ, USA
[*]Correspondence: Justin K. Sheen, jsheen@princeton.edu

January 15, 2025

Appendix A: Additional Figures and Tables

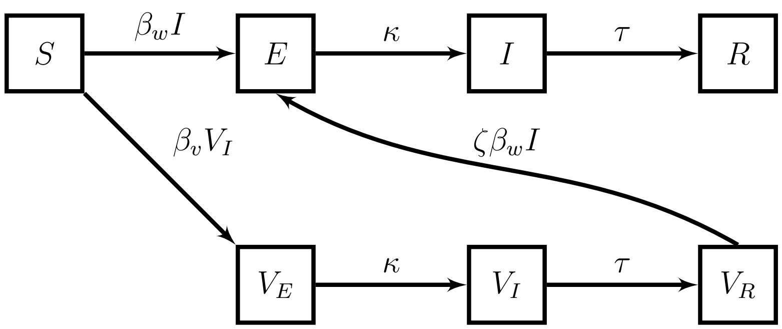

Fig A: **SEIR model with vaccination.** S is the number of susceptible animals, E is the number of infected but not infectious animals, I is the number of infectious animals, R is the number of recovered animals with immunity, V_E is the number of animals infected with the vaccinated strain who cannot transmit the vaccinated strain, V_I is the number of animals infected with vaccinated strain who can transmit the vaccinated strain, and V_R is the number of vaccinated animals with immunity. β_w is the rate of transmission of the wildtype strain, β_v is the rate of transmission of the vaccine strain, κ is 1 / the incubation period, τ is 1 / the infectious period, and ζ is 1−the efficacy of the vaccine to protect vaccinated animals from wildtype infection. We assume that both vaccine-derived and natural immunity last for the duration of the trial.

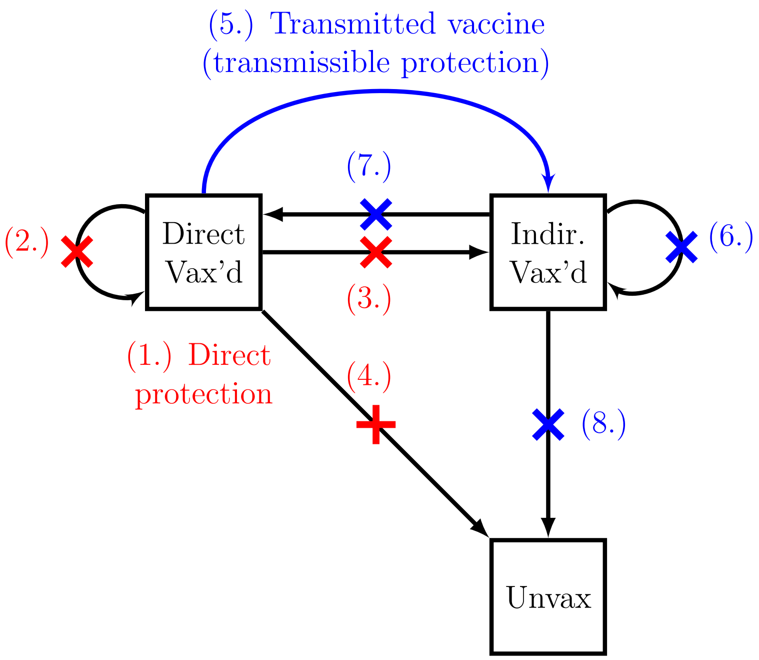

Legend:
×: Blocking of transmission
Red: protection from traditional vaccine
Blue: protection unique to transmissible vaccine

Fig B: **Transmissible vaccine protections broken down by individual.** A transmissible vaccine gives the same direct protection and 'blocking protection' (from blocking chains of transmission) given by a traditional vaccine, in red, as well as forms of protection unique to transmissible vaccines such as protection to indirectly vaccinated animals (i.e., transmissible protection) and blocking protections. Note that the transmissible protection includes the protection conferred when indirectly vaccinated animals transmit the vaccine to other initially unvaccinated animals. Directly vaccinated animals are those animals that are initially vaccinated by investigators for the trial, and indirectly vaccinated animals are those animals that become vaccinated via transmission of the vaccine from either directly vaccinated animals or other indirectly vaccinated animals. Note that the two self-referential arrows indicating blocking protections for directly vaccinated (indirectly vaccinated) animals refer to blocked transmission between directly vaccinated (indirectly vaccinated) animals.

In total, there are eight forms of protection (Fig B):

1. Vaccinal protection to directly vaccinated
2. Blocking protection from directly vaccinated to other directly vaccinated
3. Blocking protection from directly vaccinated to indirectly vaccinated
4. Blocking protection from directly vaccinated to unvaccinated
5. Transmissible protection to indirectly vaccinated (also includes protection from indirectly vaccinated to other indirectly vaccinated if there is subsequent transmission of the vaccine).
6. Blocking protection from indirectly vaccinated to other indirectly vaccinated
7. Blocking protection from indirectly vaccinated to directly vaccinated
8. Blocking protection from indirectly vaccinated to unvaccinated

Param.	Description	Value	Reference
β_w	Transmission rate of wildtype infection	Solved such that $\mathcal{R}_0$ is 2	Assumed to be highly transmissible
β_v	Transmission rate of vaccine infection	Solved such that $\mathcal{R}_0$ is 0, 0.9, 1.1	Nusimer et al.[1]
κ	1 / average incubation period	1/5 days	Chukwudi et al.[2]
τ	1 / average infectious period	1/10 days	Brown and Bevins[3]
ζ	1−Vaccine efficacy	0.2 (leaky) or 0	Miller et al.[4]

Table A: **Epidemiological parameter values.** Values and references for parameters used to simulate the epidemic model illustrative of an acute infectious poultry pathogen such as Newcastle Disease Virus. β_w and β_v are solved using the "estimate_R0" function of the *EoN* Python package for a given contact structure, β_w or β_v, and τ.[5]

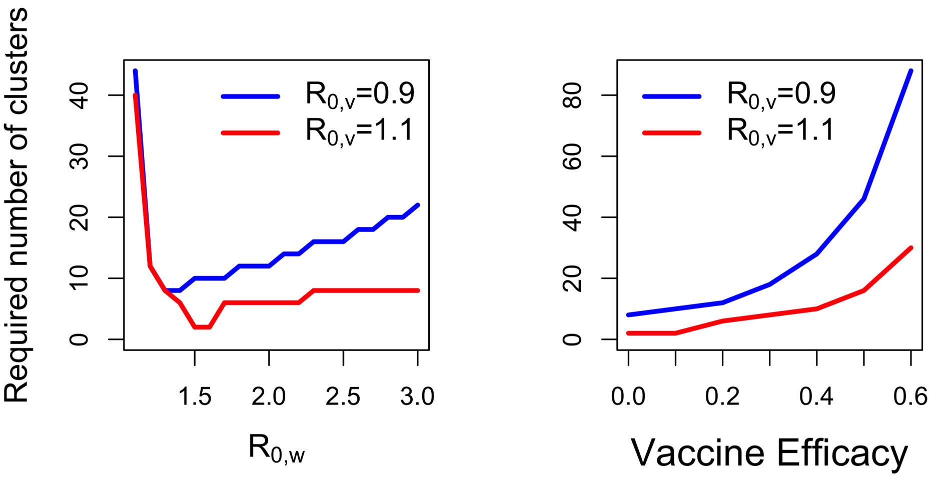

Fig C: **Required sample sizes, N_T^*, depend on $\mathcal{R}_0$ of the wildtype pathogen as well as the vaccine efficacy.** Left panel: as $\mathcal{R}_{0,w}$ increases, the required number of clusters, N_T^*, first decreases, since larger exposure of the pathogen will highlight differences in protection conferred by transmissible vs. traditional vaccines. N_T^* eventually increases; while the proportion of infected animals increases in both scenarios (transmissible vs. traditional vaccine), the difference between the two proportions decreases. Vaccine efficacy = 80%. Right panel: as vaccine efficacy decreases, N_T^* increases. All other parameters are set at number sampled from each cluster = 100, between cluster variance = 0.01, proportion initially recovered = 0, initial vaccinated proportion = 5%.

Δ	Vax. Eff.	$\mathcal{R}_{0,v}$	Estimate: mean <CI>	Req. sample size, N_T^*	Power	Type I
$\Delta_1(5)$	1	0.9	30% <29%>	28 clus., 2800 animals	80%	5%
		1.1	45% <32%>	16 clus., 1600 animals	86%	5%

Table B: **Estimated overall protection from and required sample sizes, N_T^*, for trials comparing a transmissible vaccine to a traditional vaccine when cluster size, n=500.** Prior to the outbreak, α = 5% of each cluster is vaccinated with either a transmissible or traditional, non-transmissible vaccine. Due to the smaller cluster size of 500 rather than 1000, the number of imported initial infections is 2 rather than 4 in the main results. The contact structure is Poisson distributed and trials are anticipatory. $\Delta_1(5)$ is the overall protection. Vax. Eff. is the efficacy of the vaccine to decrease susceptibility of vaccinated animals. $\mathcal{R}_{0,v}$ refers to the $\mathcal{R}_0$ of the vaccine. Estimate is the mean estimate of the overall protection comparing transmissible vaccines to traditional vaccines. $< . >$ indicates the mean 95% confidence interval width. Req. sample size, N_T^*, is the number of clusters and number of animals required from simulation to estimate the effect in order to achieve at least 80% power. Power is the percentage of simulations with a p-value ≤ 5% across 1000 trial simulations. Type I error is the percentage of simulations with a p-value ≤ 5% across 1000 trial simulations for this sample size with a traditional vaccine.

Appendix B: Estimands and Estimators

B.1 Causal Estimand Definitions and Details

B.1.1 Potential outcomes

We use the individual-level potential outcomes counterfactual framework to define our estimands, directly following and modifying Sections 2 and 3 of Hudgens and Halloran (2008).[6] Note that individuals of these trials are animals. Hudgens and Halloran (2008) defined the overall causal effect, $\overline{CE}^O$, and indirect causal effect, $\overline{CE}^I$, when there is a single possible vaccine, and we modify this framework for the case when there are two possible vaccines: a traditional, non-transmissible vaccine (trad.) or a transmissible vaccine (trans.). We also introduce an additional causal estimand to capture the causal effect based on vaccine type, $\overline{CE}^V$.

Following Section 2.1 of Hudgens and Halloran (2008), suppose there are $N > 1$ groups of individuals. For $i = 1, \ldots, N$, let n_i denote the number of individuals in group i and let $\boldsymbol{Z}_i \equiv (Z_{i1}, \ldots, Z_{in_i})$ denote the vector of treatments that the n_i individuals receive. Z_{ij} is a random variable with values 0 (not initially vaccinated), 1 (initially vaccinated with traditional vaccine), or 2 (initially vaccinated with transmissible vaccine), such that $\boldsymbol{Z}_i$ can take on 3^{n_i} possible values. Let $\boldsymbol{Z}_{i(j)}$ denote the $n_i - 1$ subvector of $\boldsymbol{Z}_i$ with the jth entry deleted. $\boldsymbol{Z}_i$ will be referred to as an intervention or treatment *program* to distinguish it from the individual treatment Z_{ij}. $\boldsymbol{z}_i$ and z_{ij} will denote possible values of $\boldsymbol{Z}_i$ and Z_{ij}.

Let R^j be the set of vectors of possible treatment programs of length j for $j = 1, 2, \ldots$; for example, $R^2 \equiv \{(0,0),(0,1),(0,2),(1,0),(1,1),(1,2),(2,0),(2,1),(2,2\}$. Possible values $\boldsymbol{z}_i$ of $\boldsymbol{Z}_i$ are elements of R^{n_i}. For positive integers n, $k \in \{0, \ldots, n\}$ and $l \in \{0, \ldots, n-k\}$, define $R^n_{k,l}$ to be the subset of R^n wherein exactly k individuals receive treatment 1 (vaccinated with traditional vaccine), l individuals receive treatment 2 (vaccinated with transmissible vaccine), and $n - k - l$ do not receive any treatment (unvaccinated). Denote the potential outcome of individual j in group i under treatment $\boldsymbol{z}_i$ as $Y_{ij}(\boldsymbol{z}_i)$.

B.1.2 Treatment assignment strategies

Following Section 2.2 of Hudgens and Halloran (2008), let ψ and ϕ denote parameterizations that determine the assignments of Z_{ij} and thus $\boldsymbol{Z}_i$ for any $i = 1, \ldots, N$. For example, in our scenario, ψ corresponds to randomly assigning five percent of animals in a group to be vaccinated with a traditional, non-transmissible vaccine (treatment 1) and ϕ might correspond to randomly assigning five percent of animals in a group to be vaccinated with a transmissible vaccine (treatment 2). ψ and ϕ are referred to as *individual treatment assignment strategies*, and we wish to identify causal effects of assigning groups to ψ or ϕ.

We consider a two-stage randomization procedure. In the first stage, each of the N groups is randomly assigned to either ψ or ϕ. In the second stage, individuals are randomly assigned treatment conditional on their group's assignment in the first stage. Corresponding to the first stage of randomization, let $\boldsymbol{S} \equiv (S_1, \ldots, S_N)$ denote the group assignments with $B_i = 0$ if the ith group is assigned to ψ and 1 if assigned to ϕ. Let ν denote the parameterization that governs the distribution of $\boldsymbol{S}$ and let $C \equiv N - \sum_i B_i$ denote the number of groups assigned

to ψ. Define ν to be a mixed or permutation group assignment strategy if $0 < C < N$ and $Pr_\nu(\boldsymbol{S} = \boldsymbol{s}) = C!(N - C)!/N!$ if $\boldsymbol{s} \in R^N_{C,0}$, 0 otherwise. In other words, under a mixed group assignment strategy, a fixed number C of N groups are assigned ψ with each of the $\binom{N}{C}$ possible group assignments receiving equal probability.

Similarly, corresponding to the second stage of randomization, let $K_i \equiv \sum_j I[Z_{ij} = 1]$, and let $L_i \equiv \sum_j I[Z_{ij} = 2]$. In other words, K_i is the sum of individuals who receive treatment 1 and L_i is the sum of individuals who receive treatment 2. Define ψ and ϕ to be mixed individual group assignment strategies if (1) K_i and L_i is fixed given B_i, with $0 \le K_i \le n_i$ and $0 \le L_i \le n_i - K_i$, i.e., given the assignment of i to ψ or ϕ, the sum of number of individuals assigned to treatment 1 or 2 is fixed, and (2) each of the $\binom{n_i}{K_i}\binom{n_i - K_i}{L_i}$ possible individual treatment assignments has equal probability of being realized for cluster i.

B.1.3 Causal estimands

Following Section 3.1 of Hudgens and Halloran (2008), the potential outcomes for individual j in group i under $z_{ij} = z$ is:

$$Y_{ij}(\boldsymbol{z}_{i(j)}, z_{ij} = z)$$

for z=0,1,2. Define the *individual average potential outcome* under treatment assignment z by:

$$\overline{Y}_{ij}(z;\psi) \equiv \sum_{\boldsymbol{\omega} \in R^{n_i - 1}} Y_{ij}(\boldsymbol{z}_{i(j)} = \boldsymbol{\omega}, z_{ij} = z) \times Pr_\psi(\boldsymbol{Z}_{i(j)} = \boldsymbol{\omega} | Z_{ij} = z)$$

This is the conditional expectation of Y_{ij} given z under assignment strategy ψ. In other words this is the conditional expectation of Y_{ij} given z across all possible assignments of all other individuals of group i. Note that depending on assignment strategy ψ, the probability that $\boldsymbol{Z}_{i(j)} = \boldsymbol{\omega}$ could be 0, for example in the extreme case if ψ assigns all individuals to treatment 1, for $\overline{Y}_{ij}(z = 1;\psi)$, all $\omega \in R^{n_i - 1}$ that do not assign everyone else in group i to treatment 1 will have probability 0.

Define the *group average potential outcome* under treatment assignment z as:

$$\overline{Y}_i(z;\psi) \equiv \frac{\sum_{j=1}^{n_i} \overline{Y}_{ij}(z;\psi)}{n_i}$$

Which is simply the average of the individual average potential outcomes across all individuals of group i. Finally define the *population average potential outcome* under treatment assignment z as:

$$\overline{Y}(z;\psi) \equiv \frac{\sum_{i=1}^{N} \overline{Y}_i(z;\psi)}{N}$$

Which is simply the average of the group average potential outcomes across all groups.

We can also define average potential outcomes solely as a function of ψ (excluding the dependence of $z_{ij} = z$). For example, define the ***marginal*** *individual average potential outcome* by $\overline{Y}_{ij}(\psi) \equiv \sum_{\boldsymbol{z} \in R^{n_i}} Y_{ij}(\boldsymbol{z}) Pr_\psi(\boldsymbol{Z}_i = \boldsymbol{z})$, that is, the average potential outcome for individual j in group i when group i is assigned ψ. The marginal group and population average potential outcomes are $\overline{Y}_i(\psi) \equiv \sum_{j=1}^{n_i} \overline{Y}_{ij}(\psi)/n_i$ and $\overline{Y}(\psi) \equiv \sum_{i=1}^{N} \overline{Y}_i(\psi)/N$, respectively.

Vaccine causal effect We define the *individual vaccine causal effect* of treatment program $\boldsymbol{z}_i$ compared with $\boldsymbol{z'}_i$ on individual j in group i is:

$$CE^V_{ij}(\boldsymbol{z}_{i(j)}, \boldsymbol{z'}_{i(j)}) \equiv Y_i(\boldsymbol{z}_{i(j)}, z_{ij} = 1) - Y_i(\boldsymbol{z'}_{i(j)}, z'_{ij} = 2)$$

where $\boldsymbol{z}_i$ is a vector of treatment assignments with $z_{ij} = 1$ and $\boldsymbol{z'}_i$ is another n_i-dimensional vector of individual treatment assignments with $z'_{ij} = 2$.

The *individual average vaccine causal effect* is:

$$\overline{CE}^V_{ij}(\psi, \phi) \equiv \overline{Y}_{ij}(1; \psi) - \overline{Y}_{ij}(2; \phi)$$

Which is the difference in individual average potential outcomes when $z_{ij} = 1$ under ψ and when $z_{ij} = 2$ under ϕ.

The *group average vaccine causal effect* is:

$$\overline{CE}^V_i(\psi, \phi) \equiv \overline{Y}_i(1; \psi) - \overline{Y}_i(2; \phi) = \sum_{j=1}^{n_i} \overline{CE}^V_{ij}(\psi, \phi)/n_i$$

The *population average vaccine causal effect* is:

$$\overline{CE}^V(\psi, \phi) \equiv \overline{Y}(1; \psi) - \overline{Y}(2; \phi) = \sum_{i=1}^{N} \overline{CE}^V_i(\psi, \phi)/N$$

Note that for the vaccine causal effect the individual treatment z and treatment assignment strategy both change depending on the arm of the contrast, and this is similar to the total effect defined previously,[6] although in contrast here we compare individual treatments that are both vaccines but of different types. We note however that the individual treatment assignment of initial vaccination with either a traditional, non-transmissible (z=1) or transmissible vaccine (z=2) are comparable since they refer to being initially vaccinated.

Indirect causal effect The *individual indirect causal effect* of treatment program $\boldsymbol{z}_i$ compared with $\boldsymbol{z'}_i$ on individual j in group i is:

$$CE^I_{ij}(\boldsymbol{z}_{i(j)}, \boldsymbol{z'}_{i(j)}) \equiv Y_i(\boldsymbol{z}_{i(j)}, z_{ij} = 0) - Y_i(\boldsymbol{z'}_{i(j)}, z'_{ij} = 0)$$

where $pmbz_i$ and $\boldsymbol{z'}_i$ are n_i-dimensional vectors of individual treatment assignments.

The *individual average indirect causal effect* is:

$$\overline{CE}^I_{ij}(\psi, \phi) \equiv \overline{Y}_{ij}(0; \psi) - \overline{Y}_{ij}(0; \phi)$$

The *group average indirect causal effect* is:

$$\overline{CE}^I_i(\psi, \phi) \equiv \overline{Y}_i(0; \psi) - \overline{Y}_i(0; \phi) = \sum_{j=1}^{n_i} \overline{CE}^I_{ij}(\psi, \phi)/n_i$$

The *population average indirect causal effect* is:

$$\overline{CE}^I(\psi, \phi) \equiv \overline{Y}(0; \psi) - \overline{Y}(0; \phi) = \sum_{i=1}^{N} \overline{CE}^I_i(\psi, \phi)/N$$

Overall causal effect: We define the *individual overall causal effect* of treatment $\boldsymbol{z}_i$ compared to treatment $\boldsymbol{z'}_i$ for individual j in group i as:

$$CE^O_{ij}(\boldsymbol{z}_i, \boldsymbol{z'}_i) \equiv Y_{ij}(\boldsymbol{z}_i) - Y_{ij}(\boldsymbol{z'}_i)$$

For the comparison of ϕ to ψ, define the *individual average overall causal effect* as:

$$\overline{CE}^O_{ij}(\psi, \phi) \equiv \overline{Y}_{ij}(\psi) - \overline{Y}_{ij}(\phi)$$

The *group average overall causal effect* is:

$$\overline{CE}^O_i(\psi, \phi) \equiv \overline{Y}_i(\psi) - \overline{Y}_i(\phi)$$

The *population average overall causal effect* is:

$$\overline{CE}^O(\psi, \phi) \equiv \overline{Y}(\psi) - \overline{Y}(\phi)$$

B.2 Estimators of causal estimands and unbiasedness, consistency, and asymptotic normality proofs

Section 4 of Hudgens and Halloran (2008) proves the unbiasedness of the following estimators in the case that there are two possible treatments, 0 and 1.[6] We check that the proofs hold in the case of a third possible treatment 2. First, we state the following assumption:

Assumption 1: ν, ϕ, ψ are mixed assignment strategies, defined in Section B.1.2

Then define the estimator (for $z = 0, 1, 2$):

$$\widehat{Y}_i(z;\psi) \equiv \frac{\sum_{j=1}^{n_i} Y_{ij}(\boldsymbol{Z}_i) I[Z_{ij} = z]}{\sum_{j=1}^{n_i} I[Z_{ij} = z]} \tag{1}$$

Theorem 1: Under Assumption 1, $E[\widehat{Y}_i(z;\psi)|B_i = 0] = \overline{Y_i}(z;\psi)$ for $z = 0, 1, 2$.
Proof: Following Proof A.1 of Hudgens and Halloran (2008), without loss of generality, let $z = 1$. Under Assumption 1, K_i and L_i are fixed, so that:

$$E[\widehat{Y}_i(1;\psi)|B_i = 0] = \frac{1}{K_i} \sum_{j=1}^{n_i} \sum_{\boldsymbol{z} \in R^{n_i}_{K_i, L_i}} Pr_\psi(\boldsymbol{Z}_i = \boldsymbol{z}) Y_{ij}(\boldsymbol{z}) I[z_{ij} = 1]$$

Any $\boldsymbol{z}$ such that $z_{ij} = 0$ or $z_{ij} = 2$ does not contribute to the summation, so that we can equivalently write:

$$\begin{aligned} E[\widehat{Y}_i(1;\psi)|B_i = 0] &= \frac{1}{K_i} \sum_{j=1}^{n_i} \sum_{\boldsymbol{\omega} \in R^{n_i-1}_{K_i-1, L_i}} Pr_\psi(\boldsymbol{Z}_{i(j)} = \boldsymbol{\omega}, Z_{ij} = 1) \times Y_{ij}(\boldsymbol{z}_{i(j)} = \boldsymbol{\omega}, z_{ij} = 1) \\ &= \frac{1}{K_i} \sum_{j=1}^{n_i} \sum_{\boldsymbol{\omega} \in R^{n_i-1}_{K_i-1, L_i}} Pr_\psi(\boldsymbol{Z}_{i(j)} = \boldsymbol{\omega} | Z_{ij} = 1) \times Pr_\psi(Z_{ij} = 1) Y_{ij}(\boldsymbol{z}_{i(j)} = \boldsymbol{\omega}, z_{ij} = 1) \end{aligned}$$

Under Assumption 1, $Pr_\psi(Z_{ij} = 1) = K_i/n_i$, implying:

$$E[\widehat{Y}_i(1;\psi)|B_i = 0] = \frac{1}{n_i}\sum_{j=1}^{n_i}\sum_{\boldsymbol{\omega}\in R^{n_i-1}_{K_i-1,L_i}} Pr_\psi(\boldsymbol{Z}_{i(j)}|Z_{ij} = 1) \times Y_{ij}(\boldsymbol{z}_{i(j)} = \boldsymbol{\omega}, z_{ij} = 1)$$
$$= \overline{Y}_i(1;\psi),$$

as desired. ■
Next define (for $z = 0,1,2$):

$$\widehat{Y}(z;\psi) \equiv \frac{\sum_{i=1}^{N}\widehat{Y}_i(z;\psi)I[B_i = 0]}{\sum_{i=1}^{N} I[B_i = 0]} \tag{2}$$

Theorem 2: Under Assumption 1, $E[\widehat{Y}(z;\psi)] = \overline{Y}(z;\psi)$ for $z = 0,1,2$.
Proof: Following Proof A.2 of Hudgens and Halloran (2008), without loss of generality, let $z = 1$. Because $E[\widehat{Y}(1;\psi)] = E[E[\widehat{Y}(1;\psi)|\boldsymbol{S}]]$, from Theorem 1 it follows that $E[\widehat{Y}(1;\psi)] = E[\sum_{i=1}^{N}\overline{Y}_i(1;\psi)I[B_i = 0]/C] = \overline{Y}(1;\psi)$.■

Thus, since $\widehat{Y}(z;\psi)$ is an unbiased estimator of $\overline{Y}(z;\psi)$, $\widehat{CE}^V(\psi,\phi) \equiv \widehat{Y}(1;\psi) - \widehat{Y}(2;\phi)$ is an unbiased estimator of the population average vaccine causal effect estimand $\overline{CE}^V(\psi,\phi)$, and $\widehat{CE}^I(\psi,\phi) \equiv \widehat{Y}(0;\psi) - \widehat{Y}(0;\phi)$ is an unbiased estimator of the population average indirect causal effect estimand $\overline{CE}^I(\psi,\phi)$.

Finally, define:

$$\widehat{Y}_i(\psi) \equiv \frac{\sum_{j=1}^{n_i} Y_{ij}(\boldsymbol{Z_i})}{n_i} \tag{3}$$
$$\widehat{Y}(\psi) \equiv \frac{\sum_{i=1}^{N}\widehat{Y}_i(\psi)I[B_i = 0]}{\sum_{i=1}^{N} I[B_i = 0]} \tag{4}$$

$\widehat{CE}^O(\psi,\phi) \equiv \widehat{Y}(\psi) - \widehat{Y}(\phi)$ was proven to be an unbiased estimator of the population average overall causal effect estimand, $\overline{CE}^O(\psi,\phi)$ in Hudgens and Halloran (2008).

B.2.1 Restrictions of causal estimands and estimators to single vaccine type per treatment assignment strategy and constant coverage level across treatment assignment strategies

Although we define the above causal estimands for any two treatment assignment strategies, ψ and ϕ, in practice we are solely interested in causal estimands where both treatment assignment strategies vaccinate the same percentage, α%, of animals of the cluster, but where one treatment assignment strategy assigns the α% to treatment 1 (traditional, non-transmissible vaccine) and the rest to treatment 0, and the other treatment assignment strategy assigns the α% to treatment 2 (transmissible vaccine) and the rest to treatment 0. Hereafter, ψ is defined as the former treatment assignment strategy (either treatment 1 or 0, i.e., the traditional vaccine arm of the trial), and ϕ is defined as the latter treatment assignment strategy (either treatment 2 or 0, i.e., the transmissible vaccine arm of the trial). Because of the dependencies on α they can be written as $\psi(\alpha)$ and $\phi(\alpha)$. We

define the restricted versions of the causal estimands (which restrict possible ψ and ϕ) as $m = \Delta_1(\alpha)$, $\Delta_2(\alpha)$, and $\Delta_3(\alpha)$ in order to avoid confusion with their unrestricted causal estimand analogues: $\overline{CE}^O$, $\overline{CE}^V$ and $\overline{CE}^I$, respectively.[1]

Next, we define estimators of $\Delta_1(\alpha)$, $\Delta_2(\alpha)$, and $\Delta_3(\alpha)$ using variables $T_{m,\ell,i}$ and $K_{m,\ell,i}$ for clearer epidemiological interpretations. $K_{m,\ell,i}$ is the number of animals in some population of interest for cluster i for estimand m of treatment arm ℓ when $\alpha\%$ is vaccinated (see below Table), where $\ell = 0$, if cluster i is assigned to $\psi(\alpha)$ and synonymously $B_i = 0$ and $\ell = 1$ if cluster i is assigned to $\phi(\alpha)$ and synonymously $B_i = 1$. $T_{m,\ell,i}$ is the number of animals of $K_{m,\ell,i}$ that are infected with the wildtype pathogen. Let N be the number of clusters of the total number of clusters of the trial, N_0, assigned to $B_i = 0$ and N_1 be the number of clusters of N assigned to $B_i = 1$, where $N_0 + N_1 = N$.

Note that hereafter we suppress the dependence on α for ease of notation, e.g., $\Delta_1(\alpha)$ will be written as Δ_1, and $\psi(\alpha)$ and $\phi(\alpha)$ will be written as ψ and ϕ, respectively.

The estimator for Δ_1 can be written as (based on the estimator of $\widehat{CE}^O$):

$$\begin{aligned}\widehat{\Delta_1} &\equiv \widehat{Y}(\psi) - \widehat{Y}(\phi)\\ &\equiv \frac{\sum_{i=1}^N \widehat{Y_i}(\psi) I[B_i = 0]}{\sum_{i=1}^N I[B_i = 0]} - \frac{\sum_{i=1}^N \widehat{Y_i}(\phi) I[B_i = 1]}{\sum_{i=1}^N I[B_i = 1]}\\ &= \frac{\sum_{i=1}^N (\sum_{j=1}^{n_i} Y_{ij}(\boldsymbol{Z_i})/n_i) I[B_i = 0]}{\sum_{i=1}^N I[B_i = 0]} - \frac{\sum_{i=1}^N (\sum_{j=1}^{n_i} Y_{ij}(\boldsymbol{Z_i})/n_i) I[B_i = 1]}{\sum_{i=1}^N I[B_i = 1]}\end{aligned}$$

Using the above definitions of $T_{m,\ell,i}$, $K_{m,\ell,i}$, N_0, and N_1:

$$\widehat{\Delta_1} = \frac{\sum_{u=1}^{N_0} (T_{m=\Delta_1,\ell=0,i=g(u)}/K_{m=\Delta_1,\ell=0,i=g(u)})}{N_0} - \frac{\sum_{u=1}^{N_1} (T_{m=\Delta_1,\ell=1,i=h(u)}/K_{m=\Delta_1,\ell=1,i=h(u)})}{N_1}$$

Where the functions $g(u)$ and $h(u)$ map the cluster index within each arm (from $u = 1$ to $u = N_0$ or N_1 for the traditional vaccine or transmissible vaccine arm, respectively) to the general cluster index, i. Next, based on the estimators of $\widehat{CE}^V$ $(\widehat{CE}^I)$, and using the definitions of $T_{m,\ell,i}$, $K_{m,\ell,i}$, N_0, and N_1 above, the estimators for $\Delta_2(\Delta_3)$ can be written as the following:

$$\begin{aligned}\widehat{\Delta_2}(\widehat{\Delta_3}) &\equiv \widehat{Y}(1(0);\psi) - \widehat{Y}(2(0);\phi)\\ &\equiv \frac{\sum_{i=1}^N \widehat{Y_i}(1(0);\psi) I[B_i = 0]}{\sum_{i=1}^N I[B_i = 0]} - \frac{\sum_{i=1}^N \widehat{Y_i}(2(0);\psi) I[B_i = 1]}{\sum_{i=1}^N I[B_i = 1]}\\ &= \frac{\sum_{i=1}^N \frac{\sum_{j=1}^{n_i} Y_{ij}(\boldsymbol{Z}_i) I[Z_{ij}=1(0)]}{\sum_{j=1}^{n_i} I[Z_{ij}=1(0)]} I[B_i = 0]}{\sum_{i=1}^N I[B_i = 0]} - \frac{\sum_{i=1}^N \frac{\sum_{j=1}^{n_i} Y_{ij}(\boldsymbol{Z}_i) I[Z_{ij}=2(0)]}{\sum_{j=1}^{n_i} I[Z_{ij}=2(0)]} I[B_i = 1]}{\sum_{i=1}^N I[B_i = 1]}\end{aligned}$$

[1] In other words we do not further consider treatment assignment strategies that assign both traditional and transmissible vaccines to animals, nor causal estimands where the percent initially vaccinated, α differs between treatment assignment strategies. Future work may seek to extend our work to consider these other treatment assignment strategies.

$$= \frac{\sum_{u=1}^{N_0} \frac{T_{m=\Delta_2(\Delta_3),\ell=0,i=g(u)}}{K_{m=\Delta_2(\Delta_3),\ell=0,i=g(u)}}}{N_0} - \frac{\sum_{u=1}^{N_1} \frac{T_{m=\Delta_2(\Delta_3),\ell=1,i=h(u)}}{K_{m=\Delta_2(\Delta_3),\ell=1,i=h(u)}}}{N_1}$$

See the Table below for a summary of the estimators of the causal estimands and their populations under consideration, where n is the cluster size, α is the proportion initially vaccinated in both arms.

Estimand (m)	Description	K
Δ_1	Overall protection	Total population, n
Δ_2	Vaccinated protection	Initially vaccinated, $n\alpha$
Δ_3	Indirect protection	Not initially vaccinated, $n - n\alpha$

B.2.2 Simple Random Sample Estimators

In practice, $\widehat{Y_i}(z,\psi)$ and $\widehat{Y_i}(\psi)$, which are used in the $\widehat{\Delta_1}$, $\widehat{\Delta_2}$, and $\widehat{\Delta_3}$ estimators, are sample means from simple random samples drawn without replacement from the n_i individuals with $Z_{ij} = z$ for $z = 0,1,2$. In any trial, we sample s animals within each cluster, i. Let $S_{m,\ell,i}$ be the number of these sampled animals that are from the population under consideration, $K_{m,\ell,i}$ (as defined previously), for estimand m (within cluster i in treatment arm ℓ) and $Z_{m,\ell,i}$ the number that are within that population and infected with wildtype infection (thus, this is a sample of $T_{m,\ell,i}$, as defined previously). Then the estimated risk in cluster i in treatment arm ℓ is $\frac{Z_{m,\ell,i}}{S_{m,\ell,i}}$. Treating $T_{m,\ell,i}$ and $K_{m,\ell,i}$ as fixed for cluster i:

$$\begin{aligned} S_{m,\ell,i} &\sim HGeom(n_i, K_{m,\ell,i}, s) \\ Z_{m,\ell,i}|S_{m,\ell,i} &\sim HGeom(K_{m,\ell,i}, T_{m,\ell,i}, S_{m,\ell,i}) \\ E[Z_{m,\ell,i}|S_{m,\ell,i}] &= T_{m,\ell,i}\frac{S_{m,\ell,i}}{K_{m,\ell,i}} \\ E\left[\frac{Z_{m,\ell,i}}{S_{m,\ell,i}}\right] &= E\left[E\left[\frac{Z_{m,\ell,i}}{S_{m,\ell,i}}|S_{m,\ell,i}\right]\right] = E\left[T_{m,\ell,i}\frac{S_{m,\ell,i}}{K_{m,\ell,i}}\frac{1}{S_{m,\ell,i}}\right] = E\left[\frac{T_{m,\ell,i}}{K_{m,\ell,i}}\right] \end{aligned}$$

The estimated risk in treatment arm ℓ is the average across all clusters in treatment arm ℓ, which is (using the definitions of N_0 and N_1): $\frac{1}{N_{0(1)}} \sum_{u=1}^{N_{0(1)}} \frac{Z_{m,\ell=0(1),i=g(u)(h(u))}}{S_{m,\ell=0(1),i=g(u)(h(u))}}$. The estimator, when a simple random sample is taken, is given by:

$$\widehat{m} = \frac{\sum_{u=1}^{N_0} \frac{Z_{m,0,i=g(u)}}{S_{m,0,i=g(u)}}}{N_0} - \frac{\sum_{u=1}^{N_1} \frac{Z_{m,1,i=h(u)}}{S_{m,1,i=h(u)}}}{N_1}$$

Thus, defining the estimators using a simple random sample of individuals in each cluster does not affect the expectation, and these simple random sample estimators remain unbiased for their respective estimands.

Consistency of Simple Random Sample Estimators Proof

We follow the consistency proof in Tsun (2021).[7] Given the estimator, $\widehat{m}$,

$$\widehat{m} = \frac{\sum_{u=1}^{N_0} \frac{Z_{m,0,i=g(u)}}{S_{m,0,i=g(u)}}}{N_0} - \frac{\sum_{u=1}^{N_1} \frac{Z_{m,1,i=h(u)}}{S_{m,1,i=h(u)}}}{N_1}$$

The estimator is consistent if:

$$\lim_{N\to\infty} Pr(|\widehat{m} - m| > \epsilon) = 0$$

Where N is the total number of clusters enrolled in the trial. $\widehat{m}$ was shown to be unbiased in Section B.2.2. Thus:

$$Pr(|\widehat{m} - m| > \epsilon) = Pr(|\widehat{m} - E[\widehat{m}]| > \epsilon)$$

Applying Chebyshev's inequality:

$$Pr(|\widehat{m} - E[\widehat{m}]| > \epsilon) \leq \frac{Var(\widehat{m})}{\epsilon^2}$$

Assuming all clusters are independent and thus that the covariance in the risk of infection between clusters is 0:

$$\begin{aligned}
Pr(|\widehat{m} - E[\widehat{m}]| > \epsilon) &\leq Var(\widehat{m})/\epsilon^2 \\
&\leq Var\left(\frac{\sum_{u=1}^{N_0} \frac{Z_{m,0,i=g(u)}}{S_{m,0,i=g(u)}}}{N_0} - \frac{\sum_{u=1}^{N_1} \frac{Z_{m,1,i=h(u)}}{S_{m,1,i=h(u)}}}{N_1}\right)\left(\frac{1}{\epsilon^2}\right) \\
&\leq \left[Var\left(\frac{\sum_{u=1}^{N_0} \frac{Z_{m,0,i=g(u)}}{S_{m,0,i=g(u)}}}{N_0}\right) + Var\left(\frac{\sum_{u=1}^{N_1} \frac{Z_{m,1,i=h(u)}}{S_{m,1,i=h(u)}}}{N_1}\right)\right]\left(\frac{1}{\epsilon^2}\right) \\
&\leq \left[\frac{1}{N_0^2} Var\left(\sum_{u=1}^{N_0} \frac{Z_{m,0,i=g(u)}}{S_{m,0,i=g(u)}}\right) + \frac{1}{N_1^2} Var\left(\sum_{u=1}^{N_1} \frac{Z_{m,1,i=h(u)}}{S_{m,1,i=h(u)}}\right)\right]\left(\frac{1}{\epsilon^2}\right) \\
&\leq \left[\frac{1}{N_0^2} \sum_{u=1}^{N_0} Var\left(\frac{Z_{m,0,i=g(u)}}{S_{m,0,i=g(u)}}\right) + \frac{1}{N_1^2} \sum_{u=1}^{N_1} Var\left(\frac{Z_{m,1,i=h(u)}}{S_{m,1,i=h(u)}}\right)\right]\left(\frac{1}{\epsilon^2}\right)
\end{aligned}$$

Since Var($\frac{Z_{m,0,i=g(u)}}{S_{m,0,i=g(u)}}$) is equivalent across all clusters assigned to $\ell = 0$ and Var($\frac{Z_{m,1,i=h(u)}}{S_{m,1,i=h(u)}}$) is equivalent across all clusters assigned to $\ell = 1$, let them be denoted generally as $Var(\frac{Z_{m,0}}{S_{m,0}})$ and Var($\frac{Z_{m,1}}{S_{m,1}}$), respectively:

$$\begin{aligned}
Pr(|\widehat{m} - E[\widehat{m}]| > \epsilon) &\leq \left[\frac{1}{N_0^2} N_0 Var\left(\frac{Z_{m,0}}{S_{m,0}}\right) + \frac{1}{N_1^2} N_1 Var\left(\frac{Z_{m,1}}{S_{m,1}}\right)\right]\left(\frac{1}{\epsilon^2}\right) \\
&\leq \left[\frac{1}{N_0} Var\left(\frac{Z_{m,0}}{S_{m,0}}\right) + \frac{1}{N_1} Var\left(\frac{Z_{m,1}}{S_{m,1}}\right)\right]\left(\frac{1}{\epsilon^2}\right)
\end{aligned}$$

$$\leq \left[\frac{1}{N/2} Var\left(\frac{Z_{m,0}}{S_{m,0}}\right) + \frac{1}{N/2} Var\left(\frac{Z_{m,1}}{S_{m,1}}\right)\right]\left(\frac{1}{\epsilon^2}\right)$$
$$\leq \left(\frac{2}{N}\right)\left[Var\left(\frac{Z_{m,0}}{S_{m,0}}\right) + Var\left(\frac{Z_{m,1}}{S_{m,1}}\right)\right]\left(\frac{1}{\epsilon^2}\right)$$

We take the limit $N \to \infty$. Since $Var(\frac{Z_{m,0}}{S_{m,0}})$ and $Var(\frac{Z_{m,1}}{S_{m,1}})$ are finite within-cluster variances that do not change as $N \to \infty$, it follows that:

$$\lim_{N\to\infty} Pr(|\widehat{m} - E[\widehat{m}]| > \epsilon) \leq \lim_{N\to\infty} \left(\frac{2}{N}\right)\left[Var\left(\frac{Z_{m,0}}{S_{m,0}}\right) + Var\left(\frac{Z_{m,1}}{S_{m,1}}\right)\right]\left(\frac{1}{\epsilon^2}\right) = 0$$

Thus, $\widehat{m}$ is a consistent estimator of m. ■

Asymptotic Normality of the Simple Random Sample Estimators

Given the estimator, $\widehat{m}$,

$$\widehat{m} = \frac{\sum_{u=1}^{N_0} \frac{Z_{m,0,i=g(u)}}{S_{m,0,i=g(u)}}}{N_0} - \frac{\sum_{u=1}^{N_1} \frac{Z_{m,1,i=h(u)}}{S_{m,1,i=h(u)}}}{N_1}$$

Each term is a sample mean of size N_ℓ sampled from the distribution of $\frac{Z_{m,\ell,i}}{S_{m,\ell,i}}$ for $\ell = 0, 1$. Also note that $\frac{Z_{m,\ell,i}}{S_{m,\ell,i}}$ for estimand m of arm ℓ must have finite variance since it is the fraction of infected animals of cluster i of the sampled animals of the subpopulation of interest and must be between 0 and 1, and that they are independent and identically distributed random variables (i.i.d) with expected value $\mu_\ell = E\left[\frac{Z_{m,\ell,i}}{S_{m,\ell,i}}\right] = E\left[\frac{T_{m,\ell,i}}{K_{m,\ell,i}}\right]$ and variance $\sigma_\ell^2 = Var\left(\frac{Z_{m,\ell,i}}{S_{m,\ell,i}}\right) = Var\left(\frac{T_{m,\ell,i}}{K_{m,\ell,i}}\right) + E\left[\frac{T_{m,\ell,i}(K_{m,\ell,i}-T_{m,\ell,i})}{K_{m,\ell,i}^2(K_{m,\ell,i}-1)}\left(K_{m,\ell,i} E\left[\frac{1}{S_{m,\ell,i}}|K_{m,\ell,i}, T_{m,\ell,i}\right] - 1\right)\right]$. Since these are finite quantities arising from the distribution of spread within the superpopulation of clusters, we can use the Central Limit Theorem.

By the Central Limit Theorem,[8] in the limit $N \to \infty$, treating N_1 and N_0 as in the previous section and as fixed proportions of N:

$$\frac{\sum_{u=1}^{N_{0(1)}} \frac{Z_{m,0(1),i=g(u)(h(u))}}{S_{m,0(1),i=g(u)(h(u))}} - N_{0(1)}\mu_{0(1)}}{\sqrt{N_{0(1)}\sigma_{0(1)}^2}} \sim \mathcal{N}(0,1),$$

the standard normal distribution. So in the limit as N_1 and $N_0 \to \infty$:

$$\frac{1}{N_0}\sum_{u=1}^{N_0}\left(\frac{Z_{m,0,i=g(u)}}{S_{m,0,i=g(u)}} - \mu_0\right) \dot{\sim} \mathcal{N}(0, \sigma_0^2/N_0) \quad \text{and} \quad \frac{1}{N_1}\sum_{u=1}^{N_1}\left(\frac{Z_{m,1,i=h(u)}}{S_{m,1,i=h(u)}} - \mu_1\right) \dot{\sim} \mathcal{N}(0, \sigma_1^2/N_1)$$

Since the clusters in the two arms are independent, then:

$$\frac{\sum_{u=1}^{N_0} \frac{Z_{m,0,i=g(u)}}{S_{m,0,i=g(u)}}}{N_0} - \mu_0 - \frac{\sum_{u=1}^{N_1} \frac{Z_{m,1,i=h(u)}}{S_{m,1,i=h(u)}}}{N_1} + \mu_1 \dot{\sim} \mathcal{N}\left(0, \frac{\sigma_0^2}{N_0} + \frac{\sigma_1^2}{N_1}\right)$$

And so:

$$\frac{\widehat{m} - (\mu_0 - \mu_1)}{\sqrt{\frac{\sigma_0^2}{N_0} + \frac{\sigma_1^2}{N_1}}} \dot{\sim} \mathcal{N}(0,1)$$

Also in the limit as $N_0, N_1 \to \infty$:

$$\widehat{m} \dot{\sim} \mathcal{N}(\mu_0 - \mu_1, \frac{\sigma_0^2}{N_0} + \frac{\sigma_1^2}{N_1})$$

So the estimator is asymptotically normal with expectation $m = \mu_0 - \mu_1$ and variance $\frac{\sigma_0^2}{N_0} - \frac{\sigma_1^2}{N_1}$, with the σ^2 values as defined above. ■

B.3 Statistical estimands of additional differences in subpopulation risks of infection

B.3.1 Statistical Estimands

We define two statistical estimands to estimate the difference in risk of infection for indirectly vaccinated animals (initially unvaccinated animals who become vaccinated through the transmissible vaccine) and never vaccinated animals (initially unvaccinated animals who never become vaccinated through the transmissible vaccine) of the transmissible vaccine arm of the trial ($\ell = 1$) in comparison to the initially unvacinated population of the traditional vaccine arm of the trial ($\ell = 0$). Note that these are not causal estimands of protection due to individual-level indirect vaccination or never vaccination, as indirect vaccination is not applied randomly across animals of a cluster and thus these estimands do not lend themselves to the exchangeability assumption of the potential outcomes framework. Instead, the following estimands are defined as true differences in cluster-level risks of infection between the two populations of each arm of the trial. Future work may seek to define these estimands within an exposure-mediator framework where the mediator is indirect vaccination.[9, 10]

The estimand for the difference in the cluster-level risk of infection among those who were indirectly vaccinated in the transmissible vaccine arm of the trial vs. those who were initially unvaccinated in the traditional vaccine arm of the trial is:

$$\Theta_1 \equiv \frac{\sum_{u=1}^{N_0} \frac{T_{m=\Theta_1,\ell=0,i=g(u)}}{K_{m=\Theta_1,\ell=0,i=g(u)}}}{N_0} - \frac{\sum_{u=1}^{N_1} \frac{T_{m=\Theta_1,\ell=1,i=h(u)}}{K_{m=\Theta_1,\ell=1,i=h(u)}}}{N_1} \tag{5}$$

Where $T_{m,\ell,i}$ and $K_{m,\ell,i}$ are the same as previously defined; in this case $K_{m=\Theta_1,\ell,i}$ is either the indirectly vaccinated population ($\ell = 1$) or initially unvaccinated population ($\ell = 0$) of cluster i, and $T_{m=\Theta_1,\ell,i}$ is the number infected with the wildtype pathogen of that population. Note that the underlying population of each arm of the trial is not the same (see below Table). Finally, note that the $T_{m,\ell,i}$ and $K_{m,\ell,i}$ notation referred to estimators of the causal estimands previously, but here we use them to define statistical estimands.

The estimand for the difference in the cluster-level risk of infection among those who were never vaccinated in the transmissible vaccine arm of the trial vs. those who were initially unvaccinated in the traditional vaccine arm of the trial is:

$$\Theta_2 \equiv \frac{\sum_{u=1}^{N_0} \frac{T_{m=\Theta_2,\ell=0,i=g(u)}}{K_{m=\Theta_2,\ell=0,i=g(u)}}}{N_0} - \frac{\sum_{u=1}^{N_1} \frac{T_{m=\Theta_2,\ell=1,i=h(u)}}{K_{m=\Theta_2,\ell=1,i=h(u)}}}{N_1} \tag{6}$$

The variables for the traditional vaccine arm of the trial are the same as for Θ_1. For the transmissible vaccine arm, $K_{m=\Theta_2,\ell,i}$ is the never vaccinated population, and $T_{m=\Theta_1,\ell,i}$ is the number infected with wildtype pathogen of the never vaccinated population. Note that the underlying population of each arm of the trial is not the same (see below Table).

See the Table below for a summary of the statistical estimands and their populations under consideration. Note that the previous Table gave a summary of underlying populations for the estimators of the causal estimands, whereas this table gives a summary of underlying populations for the statistical estimands. Let n be the total cluster size, α be the proportion initially vaccinated in both arms, and $\mathcal{F}_v$ be the final proportion of individuals vaccinated in the transmissible vaccine arm (i.e., the final size of transmission of the vaccine).

Estimand (m)	Description	K
Θ_1	Risk difference<br>for indirectly vaccinated	Initially unvaccinated, $n - n\alpha$ (ℓ=0)<br>Indirectly vaccinated, $n\mathcal{F}_v - n\alpha$ ($\ell = 1$)
Θ_2	Risk difference<br>for never vaccinated	Initially unvaccinated, $n - n\alpha$ ($\ell = 0$)<br>Never vaccinated, $n - n\mathcal{F}_v$ ($\ell = 1$)

B.3.2 Estimator

The estimator for the statistical estimands is the same as the simple random sample estimator used to estimate the causal estimands (Section B.2.2). Using the same notation as above:

$$\widehat{\Theta}_{1(2)} = \frac{\sum_{u=1}^{N_0} \frac{Z_{\Theta_{1(2)},0,i=g(u)}}{S_{\Theta_{1(2)},0,i=g(u)}}}{N_0} - \frac{\sum_{u=1}^{N_1} \frac{Z_{\Theta_{1(2)},1,i=h(u)}}{S_{\Theta_{1(2)},1,i=h(u)}}}{N_1}$$

For estimands $m = \Theta_1, \Theta_2$. The unbiasedness, consistency, and asymptotic normality of the estimator was proven in Section B.2.2.

Appendix C: Approximate sample size formula

C.1 Approach

Our objective is to arrive at required sample sizes, N_T^*, for various estimators of a two-stage randomized trial to evaluate a transmissible vaccine. We assume a simple random sample of size s from each cluster, and identify the number of clusters needed in each arm of the trial ($D = N/2 = N_1 = N_0$) to power a t-test of the difference of proportion of infected individuals between the two arms of the trial. These use defined variances of cluster-level outcomes (proportion of individuals infected) between the two arms of the trial (Details of estimands in Appendix 2). They also rely on transmission models for both transmissible vaccine transmission and wildtype pathogen transmission, which are used to find the expected final sizes after a wildtype outbreak when (1) Scenario 1: The $\mathcal{R}_0$ of the vaccine, $\mathcal{R}_{0,v} \neq$

0 (transmissible vaccine), and (2) Scenario 2: $\mathcal{R}_{0,v} = 0$ (traditional vaccine).[2] These are implemented in an R Shiny application where users can explore the dependencies of N_T^* with various ecological and statistical factors.

C.2 Sample Size Required for *t*-Test

To test the null hypothesis of zero risk difference, $H_0 : \Delta_1, \Delta_2, \Delta_3, \Theta_1,$ or $\Theta_2 = 0$, we can use Welch's t-test comparing the means from each treatment arm, where the null hypothesis is that the two arms of the trial have equal means. Note that whether the hypothesis test tests for a statistically significant causal effect or statistical association depends on whether a causal or statistical estimand is used in the test statistic. The t-statistic is $\frac{\overline{X_1}-\overline{X_2}}{\sqrt{s^2_{\overline{X_1}}+s^2_{\overline{X_2}}}}$ where $\overline{X_1}$ and $\overline{X_2}$ are the mean proportion of infections in treatment arms 1 and 2, respectively, and $s^2_{\overline{X_1}}$ and $s^2_{\overline{X_2}}$ are the standard errors of the sample for treatment arms 1 and 2, respectively. Note that in the actual analysis, we would use an exact test such as the permutation test, but we use the t-test to get approximate sample sizes. Following Sheen et al. 2022,[11] the required number of clusters D in each arm is given by:

$$D = \frac{\left(Var\left(\frac{Z_{m,0,i}}{S_{m,0,i}}\right) + Var\left(\frac{Z_{m,1,i}}{S_{m,1,i}}\right)\right)\left(t_{2N-2,1-\alpha/2} + t_{2N-2,1-\beta}\right)}{\Delta_j^2},$$

where $t_{DF,\phi}$ is the ϕ-quantile of the t-distribution with DF degrees of freedom, with power $1-\beta$ and two-sided significance level α. We use $1-\beta = 0.8$ and $\alpha = 0.05$. The total number of required clusters is $N_T^* = 2D$ and the total number of required sampled individuals is $2Ds$.

C.3 Variance of Cluster-Level Outcomes

As shown in Appendix 2, the simple random sample estimators of either the causal estimands, $\Delta_1, \Delta_2, \Delta_3$, and estimands to estimates differences in risk of infection for subpopulations of the transmissible vaccine arm of the trial, Θ_1, Θ_2 are notated using the average of $Z_{m,\ell,i}/S_{m,\ell,i}$ across clusters, where the denominator is a sample of the population of interest for estimand m, $K_{m,\ell,i}$, and the numerator is a sample of the number of infected of the sample from $K_{m,\ell,i}$, $T_{m,\ell,i}$. To analytically estimate the required sample size, N_T^*, we need estimates of the variance of the estimated risks across clusters, $Var\left(\frac{Z_{m,\ell,i}}{S_{m,\ell,i}}\right)$. Again, in the trial itself, the bootstrap variance method would likely be preferred to avoid making specific distributional and epidemic size assumptions.

Under the same distributional assumptions as above:

$$Var(Z_{m,\ell,i}|S_{m,\ell,i}, T_{m,\ell,i}, K_{m,\ell,i}) = S_{m,\ell,i}\frac{T_{m,\ell,i}}{K_{m,\ell,i}}\frac{K_{m,\ell,i}-T_{m,\ell,i}}{K_{m,\ell,i}}\frac{K_{m,\ell,i}-S_{m,\ell,i}}{K_{m,\ell,i}-1}$$

[2]When the vaccine has no effect at reducing susceptibility of vaccinated individuals this is equivalent to a control group where no vaccination is implemented. Thus, the comparison between Scenario 1 and Scenario 2 is equivalent to comparing clusters where the transmissible vaccine was implemented vs. clusters where no vaccination was implemented.

$$Var\left(\frac{Z_{m,\ell,i}}{S_{m,\ell,i}}|S_{m,\ell,i},T_{m,\ell,i},K_{m,\ell,i}\right) = \frac{1}{S_{m,\ell,i}}\frac{T_{m,\ell,i}}{K_{m,\ell,i}}\frac{K_{m,\ell,i}-T_{m,\ell,i}}{K_{m,\ell,i}}\frac{K_{m,\ell,i}-S_{m,\ell,i}}{K_{m,\ell,i}-1}$$

$$Var(S_{m,\ell,i}|T_{m,\ell,i},K_{m,\ell,i}) = s\frac{K_{m,\ell,i}}{n}\frac{n-K_{m,\ell,i}}{n}\frac{n-s}{n-1}$$

$$\begin{aligned}Var\left(\frac{Z_{m,\ell,i}}{S_{m,\ell,i}}\right) &= E\left[Var\left(\frac{Z_{m,\ell,i}}{S_{m,\ell,i}}|S_{m,\ell,i},T_{m,\ell,i},K_{m,\ell,i}\right)\right]\\ &\quad + Var\left(E\left[\frac{Z_{m,\ell,i}}{S_{m,\ell,i}}|S_{m,\ell,i},T_{m,\ell,i},K_{m,\ell,i}\right]\right)\\ &= E\left[\frac{T_{m,\ell,i}}{K_{m,\ell,i}}\frac{K_{m,\ell,i}-T_{m,\ell,i}}{K_{m,\ell,i}}\frac{K_{m,\ell,i}-S_{m,\ell,i}}{S_{m,\ell,i}(K_{m,\ell,i}-1)}\right] + Var\left(\frac{T_{m,\ell,i}}{K_{m,\ell,i}}\right)\end{aligned}$$

There are essentially four sources of variation in this estimate: (1) the between-cluster variability of the population under consideration, $K_{m,\ell,i}$; (2) the between-cluster variability of the number infected within that population, $T_{m,\ell,i}$; (3) the sampling variability of the number of sampled individuals within the population, $S_{m,\ell,i}$; and (4) the sampling variability of the outcome among the sampled individuals.

If we assume that the number of individuals sampled within the population under consideration is fixed (i.e., $S_{m,\ell,i} = s_{m,\ell}$ by design or as a simplifying assumption) and that the size of the population under consideration is fixed within each arm (i.e., $K_{m,\ell,i} = k_{m,\ell}$ due to the population under consideration or by simplifying assumption), then the variance formula simplifies to:

$$\begin{aligned}Var\left(\frac{Z_{m,\ell,i}}{S_{m,\ell,i}}\right) &= E\left[\frac{T_{m,\ell,i}}{k_{m,\ell}}\frac{k_{m,\ell}-T_{m,\ell,i}}{k_{m,\ell}}\frac{k_{m,\ell}-s_{m,\ell}}{s_{m,\ell}(k_{m,\ell}-1)}\right] + Var\left(\frac{T_{m,\ell,i}}{k_{m,\ell}}\right)\\ &= \frac{k_{m,\ell}-s_{m,\ell}}{s_{m,\ell}(k_{m,\ell}-1)}E\left[\frac{T_{m,\ell,i}}{k_{m,\ell}}\left(1-\frac{T_{m,\ell,i}}{k_{m,\ell}}\right)\right] + Var\left(\frac{T_{m,\ell,i}}{k_{m,\ell}}\right)\\ &= \frac{k_{m,\ell}-s_{m,\ell}}{s_{m,\ell}(k_{m,\ell}-1)}\left(E\left[\frac{T_{m,\ell,i}}{k_{m,\ell}}\right]\left(1-E\left[\frac{T_{m,\ell,i}}{k_{m,\ell}}\right]\right) - Var\left[\frac{T_{m,\ell,i}}{k_{m,\ell}}\right]\right) + Var\left[\frac{T_{m,\ell,i}}{k_{m,\ell}}\right]\\ &= \frac{k_{m,\ell}-s_{m,\ell}}{s_{m,\ell}(k_{m,\ell}-1)}\left(E\left[\frac{T_{m,\ell,i}}{k_{m,\ell}}\right]\left(1-E\left[\frac{T_{m,\ell,i}}{k_{m,\ell}}\right]\right)\right) + \frac{k_{m,\ell}}{k_{m,\ell}-1}\frac{s_{m,\ell}-1}{s_{m,\ell}}Var\left(\frac{T_{m,\ell,i}}{k_{m,\ell}}\right)\end{aligned}$$

The plug-in hypothesized effect size of using a transmissible vs. a traditional vaccine will be given by:

$$E\left[\frac{T_{m,0,i}}{k_{m,0}}\right] - E\left[\frac{T_{m,1,i}}{k_{m,1}}\right] = \mathcal{F}_{0,m} - \mathcal{F}_{1,m},$$

the difference in the expected final proportion infected among the population under consideration between the two treatment arms. The final proportions are calculated according to epidemiological parameters of the outbreak (see Section C.4 and C.5).

The following subsections identify plug-in estimates and values to use in these formulae, which then inform the sample size formula given above. Note that the estimated sample size for estimating Δ_1 involves relatively few simplifying assumptions, the sample sizes for estimating Δ_2 or Δ_3 includes an additional assumption on the vaccine's effect and an additional assumption on the sampling procedure, and the sample sizes for estimating Θ_1 or Θ_2 require

yet an additional simplifying assumption. These assumptions may make the analytic power and sample size calculations less reliable; they will generally be anti-conservative because they ignore one or more of the above sources of variability.

C.3.1 Assumptions and Parameter Values for Estimator of Overall Causal Effect, Δ_1

For the estimand Δ_1, which calculates the risk difference between the overall populations from both the trad. vax and trans. vax arms, the population under consideration is the full cluster, so $K_{\Delta_1,\ell,i} = n$ and $S_{\Delta_1,\ell,i} = s$ for all ℓ, i by design. In this case, then, the variance simplifies to:

$$Var\left(\frac{Z_{\Delta_1,\ell,i}}{S_{\Delta_1,\ell,i}}\right) = \frac{n-s}{s(n-1)}\left(E\left[\frac{T_{\Delta_1,\ell,i}}{n}\right]\left(1-E\left[\frac{T_{\Delta_1,\ell,i}}{n}\right]\right)\right) + \frac{n}{n-1}\frac{s-1}{s}Var\left(\frac{T_{\Delta_1,\ell,i}}{n}\right)$$

$T_{\Delta_1,\ell,i}$ is the total number of infected individuals in cluster i in treatment arm ℓ. So $T_{\Delta_1,\ell,i}/n$ is the proportion infected within the cluster. The expectation is given by the overall final size, $\mathcal{F}_{\ell,\Delta_1} = \mathcal{F}_\ell$ (see section C.5 for details on a deterministic SEIR model that can be used for this). The variance estimator also requires an estimate of the between-cluster variability of that final size proportion, σ^2_ℓ.

C.3.2 Variance for Estimator of Vaccine Causal Effect, Δ_2

For the estimand Δ_2, which calculates the risk difference between the initially vaccinated populations from both the trad. vax and trans. vax arms, the population under consideration is the initially vaccinated population in each treatment arm. This population is fixed, so $K_{\Delta_2,\ell,i} = n\alpha$ for all ℓ, i.

In addition, if we assume that the s sampled animals are all taken from the initially vaccinated population, we have $S_{\Delta_2,\ell,i} = s$ for all ℓ, i. Note that this could be accomplished by sampling until s animals from that population are identified. If sampling is not conducted this way, the average number of animals sampled from that population can be used (i.e., use $s\alpha$ instead if s animals are sampled from the whole population), but this will tend to underestimate the variance and thus N^*_T.

The variance then simplifies to:

$$Var\left(\frac{Z_{\Delta_2,\ell,i}}{S_{\Delta_2,\ell,i}}\right) = \frac{n\alpha-s}{s(n\alpha-1)}\left(E\left[\frac{T_{\Delta_2,\ell,i}}{n\alpha}\right]\left(1-E\left[\frac{T_{\Delta_2,\ell,i}}{n\alpha}\right]\right)\right) + \frac{n\alpha}{n\alpha-1}\frac{s-1}{s}Var\left(\frac{T_{\Delta_2,\ell,i}}{n\alpha}\right)$$

$T_{\Delta_2,\ell,i}$ is the total number of infected initially vaccinated individuals in cluster i in treatment arm ℓ and $T_{\Delta_2,\ell,i}/n\alpha$ is the corresponding proportion. The variance estimator here, then, requires an estimate of the average proportion of vaccinated individuals infected $\mathcal{F}_{\ell,\Delta_2}$ for each treatment arm and an estimate of the variability of that proportion, σ^2_{ℓ,Δ_2}. These can be plugged in for $E\left[\frac{T_{\Delta_2,\ell,i}}{n}\right]$ and $Var\left(\frac{T_{\Delta_2,\ell,i}}{n}\right)$, respectively, in the estimand and variance formulae.

Using the deterministic SEIR model, see Section C.5, the final size of the epidemic among the vaccinated population can be determined for both treatment arms; denote these by $\mathcal{F}_{0,vax.}$ and $\mathcal{F}_{1,vax.}$.

Then $\mathcal{F}_{0,\Delta_2} = \frac{n\mathcal{F}_{0,vax.}}{n\alpha} = \frac{\mathcal{F}_{0,vax.}}{\alpha}$. The total vaccinated proportion for the transmissible vaccine case includes both initially-vaccinated and indirectly-vaccinated animals. We scale this by the proportion of the total vaccinated that are initially vaccinated to get the appropriate value here, under the assumption that the vaccine is equally effective in both groups. So, letting $\mathcal{F}_v$ be the final proportion of individuals vaccinated (directly and indirectly):

$$\mathcal{F}_{1,\Delta_2} = \frac{n\mathcal{F}_{1,vax.}}{n\alpha} \cdot \frac{n\alpha}{n\mathcal{F}_v} = \frac{\mathcal{F}_{1,vax.}}{\mathcal{F}_v}$$

If more precision is desired, a model that maintains different compartments for initially and indirectly vaccinated animals could be used instead.

The between-cluster variability σ^2_{ℓ,Δ_2} may be hard to estimate in this case. If the estimates $\mathcal{F}_\ell$ and variances σ^2_{ℓ,Δ_1} have already been identified for the Estimator of Δ_1, one approximation would be to scale that variance by the square of the ratio of the proportion estimate for the Estimator of Δ_2 to the proportion estimate for estimator 1: $\sigma^2_{\ell,\Delta_2} \approx \sigma^2_{\ell,\Delta_1}\left(\frac{\mathcal{F}_{\ell,\Delta_2}}{\mathcal{F}_\ell}\right)^2$.

C.3.3 Variance for Estimator of Indirect Causal Effect, Δ_3

For the estimand Δ_3, which calculates the risk difference between the initially unvaccinated populations from both the trad. vax and trans. vax arms, the results follow those for Δ_2 but on the initially unvaccinated instead of vaccinated population, with the fraction of the population being $1-\alpha$ instead of α, so $K_{\Delta_3,\ell,i} = n(1-\alpha)$ for all ℓ, i. The appropriate sampling number to use is $S_{\Delta_3,\ell,i} = s$ if s individuals from the initially unvaccinated population are sampled, or instead use $S_{\Delta_3,\ell,i} = s(1-\alpha)$ as an average if s individuals from the whole population are sampled. Similarly to Δ_2, then, we have:

$$\begin{aligned} Var\left(\frac{Z_{\Delta_3,\ell,i}}{S_{\Delta_3,\ell,i}}\right) = \frac{n(1-\alpha)-s}{s(n(1-\alpha)-1)}\left(E\left[\frac{T_{\Delta_3,\ell,i}}{n(1-\alpha)}\right]\left(1-E\left[\frac{T_{\Delta_3,\ell,i}}{n(1-\alpha)}\right]\right)\right) \\ + \frac{n(1-\alpha)}{n(1-\alpha)-1}\frac{s-1}{s}Var\left(\frac{T_{\Delta_3,\ell,i}}{n(1-\alpha)}\right) \end{aligned}$$

$T_{\Delta_3,\ell,i}$ is the total number of infected initially unvaccinated individuals in cluster i in treatment arm ℓ and $T_{\Delta_3,\ell,i}/n(1-\alpha)$ is the corresponding proportion. The variance estimator here, then, requires an estimate of the average proportion of unvaccinated individuals infected $\mathcal{F}_{\ell,\Delta_3}$ for each treatment arm and an estimate of the variability of that proportion, σ^2_{ℓ,Δ_3}. These can be plugged in for $E\left[\frac{T_{\Delta_3,\ell,i}}{n(1-\alpha)}\right]$ and $Var\left(\frac{T_{\Delta_3,\ell,i}}{n(1-\alpha)}\right)$, respectively, in the estimand and variance formulae.

Using the deterministic SEIR model, see section C.5, the final size of the epidemic among the unvaccinated population can be determined for both treatment arms; denote these by $\mathcal{F}_{0,unvax.}$ and $\mathcal{F}_{1,unvax.}$. Using the intermediate results from Δ_1 and Δ_2 (and under equivalent assumptions), we can find these by $\mathcal{F}_{\ell,unvax.} = \mathcal{F}_\ell - \mathcal{F}_{\ell,vax.}$. Then $\mathcal{F}_{0,\Delta_3} = \frac{\mathcal{F}_{0,unvax.}}{1-\alpha}$ and $\mathcal{F}_{1,\Delta_3} = \frac{\mathcal{F}_{1,unvax.}}{1-\alpha}$.

Again, the between-cluster variability σ^2_{ℓ,Δ_3} may be hard to estimate directly, so the approximation that scales σ^2_{ℓ,Δ_1} can be used: $\sigma^2_{\ell,\Delta_3} \approx \sigma^2_{\ell,\Delta_1}\left(\frac{\mathcal{F}_{\ell,\Delta_3}}{\mathcal{F}_\ell}\right)^2$.

C.3.4 Variance for Estimator of Difference in Risk of Infection for Indirectly Vaccinated, Θ_1

For the estimand Θ_1, which calculates the risk difference between the initially unvaccinated population from the trad. vax arm, and the indirectly vaccinated population of the trans. vax arm, the population under consideration is the initially unvaccinated population in the traditional vaccine arm and the indirectly vaccinated population in the transmissible vaccine arm. The plug-in estimators and parameter values for the traditional arm thus are the same as for Δ_3: $S_{\Theta_1,0,i} = s$ (or $s(1-\alpha)$), $K_{\Theta_1,0,i} = n(1-\alpha)$, $\mathcal{F}_{0,\Theta_1} = \mathcal{F}_{0,\Delta_3}$, and $\sigma^2_{0,2} = \sigma^2_{0,\Delta_3}$.

For the transmissible arm, the true $K_{\Theta_1,1,i}$ value now varies by cluster depending on the amount of transmission of the vaccine. We proceed via a simplifying assumption that ignores this variability, setting $K_{\Theta_1,1,i} = n(\mathcal{F}_v - \alpha)$ for all clusters.

In addition, there may be additional variability in the number of animals sampled from the population under consideration due to the variability in K. Again, we either remove this variability by design (and use $S_{\Theta_1,1,i} = s$) or ignore it (using the average $S_{\Theta_1,1,i} = s(\mathcal{F}_v - \alpha)$ or $S_{\Theta_1,1,i} = s\frac{(\mathcal{F}_v-\alpha)}{1-\alpha}$ depending on whether there is immediate identification of initially vaccinated from initially unvaccinated).

The number of infections among this population can then be found using the intermediate results from Δ_2 (it is in fact the same value, due to the assumption that the proportion of infections among the indirectly vaccinated will be equal to that among those directly vaccinated):

$$\mathcal{F}_{1,\Theta_1} = \frac{n\mathcal{F}_{1,vax}}{n(\mathcal{F}_v - \alpha)} \cdot \frac{n(\mathcal{F}_v - \alpha)}{\mathcal{F}_v} = \frac{\mathcal{F}_{1,vax.}}{\mathcal{F}_v}$$

The between-cluster variability can again be approximated as in the Δ_2 case: $\sigma^2_{1,\Theta_1} \approx \sigma^2_{1,\Delta_1}\left(\frac{\mathcal{F}_{1,\Theta_1}}{\mathcal{F}_{1,\Delta_1}}\right)^2$.

C.3.5 Variance for Estimator of Difference in Risk of Infection for Never Vaccinated, Θ_2

For the estimand Θ_2, which calculates the risk difference between the initially unvaccinated population from the trad. vax arm, and the never vaccinated (neither directly nor indirectly) population of the trans. vax arm, the population under consideration is the initially unvaccinated population in the traditional vaccine arm and the never-vaccinated population in the transmissible vaccine arm. The plug-in estimators and parameter values for the traditional arm again are the same as for Δ_3: $S_{\Theta_2,0,i} = s$ (or $s(1-\alpha)$), $K_{\Theta_2,0,i} = n(1-\alpha)$, $\mathcal{F}_{0,\Theta_2} = \mathcal{F}_{0,\Delta_3}$, and $\sigma^2_{0,\Theta_2} = \sigma^2_{0,\Delta_3}$.

For the transmissible arm, the true $K_{\Theta_2,1,i}$ value again varies by cluster depending on the amount of transmission of the vaccine. We proceed via a simplifying assumption that ignores this variability, setting $K_{\Theta_2,1,i} = n(1-\mathcal{F}_v)$ for all clusters.

In addition, there may again be additional variability in the number of animals sampled from the population under consideration due to the variability in K. Again, we either remove this variability by design (and use $S_{\Theta_2,1,i} = s$) or ignore it (using the average $S_{\Theta_2,1,i} = s(1-\mathcal{F}_v)$ or $S_{\Theta_2,1,i} = s\frac{(1-\mathcal{F}_v)}{1-\alpha}$ depending on whether there is immediate identification of initially vaccinated from initially unvaccinated).

The number of infections among this population can then be found using the intermediate results from Δ_1 and Δ_2:

$$\mathcal{F}_{1,\Theta_2} = \frac{\mathcal{F}_1 - \mathcal{F}_{1,vax.}}{1 - \mathcal{F}_v}$$

The between-cluster variability can again be approximated similarly to previous cases: $\sigma^2_{1,\Theta_2} \approx \sigma^2_{1,\Delta_1}\left(\frac{\mathcal{F}_{1,\Theta_2}}{\mathcal{F}_{1,\Delta_1}}\right)^2$.

C.4 Description of R Shiny Application for Analytic Sample Size Calculations

The formula for the required sample size, N_T^*, of the estimands described above are implemented in an R Shiny application available at `https://j-k-s.shinyapps.io/shinyTrans/`.

The following parameters are input by the user:

1. n: number of indivs. in each cluster
2. q: prop. of indivs. initially recovered from wildtype infection
3. I_0: prop. indivs. initially infected at start of wildtype outbreak (= $1/n$)
4. $\mathcal{R}_{0,w}$: $\mathcal{R}_0$ of wildtype pathogen
5. α: initial vaccinated proportion of entire pop
6. $\mathcal{R}_{0,t}$: $\mathcal{R}_0$ of traditional vaccine, which is 0
7. $\mathcal{R}_{0,v}$: $\mathcal{R}_0$ of transmissible vaccine
8. $\phi_{S,0(1)}$: susceptibility decrease due to vaccination (i.e. 1 - VaxEff)
   - 0 = traditional vaccine, 1 = transmissible vaccine
9. s: number to sample from each cluster

Note that the causal estimands, Δ_1, Δ_2, Δ_3, are only defined in the case $q = 0$, when there is no prior immunity. We allow $q > 0$ in the R Shiny Application for future extensions. These parameters lead to the expected final size proportions, $\mathcal{F}_{\ell,m}$, through a deterministic SEIR model, as outlined in the next section.

We then output the required number of clusters, N_T^*, to power the trial, using the formulae and simplifying assumptions described in the previous section. We model anticipatory trials, where vaccination is implemented and the transmissible vaccine is allowed to circulate before an outbreak of a wildtype pathogen. Note that the user must specify an estimated between-cluster variability of the final size proportion (i.e., for Δ_1). This variability of Δ_1 is then used to approximate the values for the other estimands, as described above.

We allows users to toggle between two scenarios of sampling: if investigators can immediately differentiate between initially vaccinated from initially unvaccinated animals easily, or not. For example, this is possible if investigators tag animals they initially vaccinate, so they avoid including initially vaccinated animals in their sample. In the case that investigators

can immediately differentiate the two groups, then we sample, s, from the sub-populations $S_{m,\ell,i}$=$n\alpha$ or $n-n\alpha$ of the relevant estimands; if not, s is sampled from the entire population n, and the sample is subset to the relevant population of the estimand.

Whether or not the differentiation is immediate or not, to estimate the risk difference between directly vaccinated animals given the traditional vaccine vs. transmissible vaccine, Δ_2, and the risk difference between initially unvaccinated animals in clusters given the traditional vaccine vs. transmissible vaccine, Δ_3 we assume investigators can tell whether an animal was directly vaccinated or not.

For estimands Θ_1 and Θ_2, which required knowing which initially unvaccinated animals were indirectly vaccinated vs. never vaccinated for the transmissible vaccine arm, we assume that investigators would not immediately know whether this was the case. Thus, we sample s from either the initially unvaccinated subgroup, or entire population (depending on the user input of whether investigators can immediately tell if an animal was initially vaccinated or unvaccinated), which implicitly samples from both the indirectly vaccinated and never vaccinated subgroups in either scenario. We then subset to the relevant sub-population of the transmissible vaccine arm of each estimand after determination of which sub-population they belong to. This determination could be done for example through serology tests that can differentiate between vaccinated and infected (DIVA) serological responses.

C.5 Identifying Deterministic Epidemic Parameters

C.5.1 Transmission model

To arrive at final size proportions of treatment arm ℓ for estimand m, $\mathcal{F}_{\ell,m}$, we use the following transmission model described by Arino et al.[12] We will use this to model transmission of the vaccine as well as transmission of the wildtype pathogen.

$$\begin{aligned}
\frac{dS}{dt} &= -\beta S[I + \phi_I I_T] \\
\frac{dS_T}{dt} &= -\phi_S \beta S[I + \phi_I I_T] \\
\frac{dL}{dt} &= \beta S_T[I + \phi_I] - \kappa_L \\
\frac{dL_T}{dt} &= \phi_S \beta S_T[I + \phi_I I_T] - \kappa_T \\
\frac{dI}{dt} &= \kappa_L - \tau I \\
\frac{dI_T}{dt} &= \kappa_T L_T - \tau_T I_T \\
\frac{dR}{dt} &= f\tau I + f_T \tau_T I
\end{aligned}$$

Where $S(0) = (1-\gamma)S_0$, $S_T(0) = \gamma S_0$, $I(0) = I_0$,$Y_{\ell,i}$ $L(0) = L_T(0) = I_T(0) = 0$. S denotes the naive susceptibles and S_T denotes the vaccinated "susceptibles" when $\phi_S < 1$ or $\phi_I < 1$. A fraction γ of susceptibles have been vaccinated before the beginning of the epidemic with a vaccine that multiples susceptibility by a factor $\phi_S \leq 1$ and infectivity by a factor $\phi_I \leq 1$.

The latent periods for unvaccinated and vaccinated individuals are $1/\kappa$ and $1/\kappa_T$, respectively. The infectious periods for unvaccinated and vaccinated individuals are $1/\tau$ and $1/\tau_T$, respectively. The percent that recover (i.e. do not die) is f for unvaccinated individuals and f_T for vaccinated individuals.

The final size relation for this model, proven in the Supplementary Material of Arino et al.,[12] is:

$$ln\Big(\frac{(1-\gamma)S_0}{S_\infty}\Big) = \frac{\beta}{\tau}[(1-\gamma)S_0 - S_\infty] + \frac{\phi_I\beta}{\tau_T}[\gamma S_0 - S_{T,\infty}] + \frac{\beta I_0}{\tau}$$
$$S_{T,\infty} = \gamma S_0\Big(\frac{S_\infty}{(1-\gamma)S_0}\Big)^{\phi_S}$$

And:

$$\mathcal{R}_c = S_0\beta\Big[\frac{1-\gamma}{\tau} + \frac{\phi_I\phi_S\gamma}{\tau_T}\Big]$$

If we assume $S_0 = 1, \tau = \tau_T$, and $\phi_I = 1$ if vaccination solely reduces susceptibility of vaccinated individuals to infection then:

$$\mathcal{R}_c = \frac{\beta}{\tau}[1-\gamma+\phi_S\gamma]$$

We define R_0 as R_c when $\gamma = 0$, i.e. there are no vaccinated individuals initially. Thus:

$$\begin{aligned}\mathcal{R}_0 &= \frac{\beta}{\tau}[1]\\ &= \frac{\beta}{\tau}\end{aligned}$$

Which is the usual value of $\mathcal{R}_0$ in the simplest SIR model. We can then substitute $\mathcal{R}_0$ into the final size relation:

$$ln\Big(\frac{(1-\gamma)S_0}{S_\infty}\Big) = \mathcal{R}_0[(1-\gamma)S_0 - S_\infty] + \mathcal{R}_0[\gamma S_0 - S_{T,\infty}] + \mathcal{R}_0 I_0$$
$$S_{T,\infty} = \gamma S_0\Big(\frac{S_\infty}{(1-\gamma)S_0}\Big)^{\phi_S}$$

$S_0 = 1 - I_0 - q$ where q is the proportion already recovered and I_0 is the starting proportion of infected individuals. Implicitly, we assume recovered individuals do not affect epidemic dynamics beyond reducing the proportion of susceptibles. The starting proportion of infected individuals is taken from the remaining population once the initially recovered proportion, q, is removed. Thus, the epidemic dynamics then take place in the population $S_0 + I_0 = 1 - q$

If we rearrange this first equation we get:

$$\mathcal{R}_0 = \frac{ln\Big(\frac{(1-\gamma)(1-I_0-q)}{S_\infty}\Big)}{[(1-\gamma)(1-I_0-q) - S_\infty] + \gamma(1-I_0-q)\Big[1-\Big(\frac{S_\infty}{(1-\gamma)(1-I_0-q)}\Big)^{\phi_S}\Big] + I_0}$$

$$\mathcal{R}_0 = f(I_0, \gamma, S_\infty, \phi_S, q)$$
$$S_\infty = f^{-1}(I_0, \gamma, \mathcal{R}_0, \phi_S, q)$$

This relation can be used to numerically evaluate the relationship between the $\mathcal{R}_0$ of a vaccine or wildtype pathogen and the final size of its transmission. The final proportion of vaccinated individuals, $S_{T,\infty}$ can be calculated based on S_∞ since S_∞, γ, and ϕ_S are known.

C.5.2 $\mathcal{F}_0$ and $\mathcal{F}_1$ for the Estimator of the Overall Causal Effect, Δ_1

For the estimand Δ_1,which calculates the risk difference between the overall populations of the trad. vax and trans. vax arms, we first calculate $\mathcal{F}_0$, the proportion of infected individuals in the entire cluster in the traditional vaccine arm who are infected:

$$S_0 = 1 - I_0 - q$$
$$S_{\infty,0} = f^{-1}(I_0 = I_0, \gamma = \frac{\alpha}{S_0}, \mathcal{R}_0 = \mathcal{R}_{0,w}, \phi_S = \phi_{S,0}, q = q)$$
$$S_{T,\infty,0} = \gamma S_0 \big(\frac{S_{\infty,0}}{(1-\gamma)S_0}\big)^{\phi_{S,0}}$$
$$\mathcal{F}_0 = 1 - S_{\infty,0} - S_{T,\infty,0}$$

Next, we calculate $\mathcal{F}_{1,\Delta_1}$, the proportion of infected individuals in the entire cluster in the transmissible vaccine arm who are infected. We first calculate a pre-step for $\mathcal{F}_{1,\Delta_1}$ by calculating the proportion either directly or indirectly vaccinated, $\mathcal{F}_v$, by simulating transmission of the transmissible vaccine:

$$\mathcal{F}_v = 1 - f^{-1}(I_0 = \alpha, \gamma = 0, \mathcal{R}_0 = \mathcal{R}_{0,v}, \phi_S = 0, q = q) - q$$

Note that for $\mathcal{F}_v$, $I_0 = \alpha$, the starting proportion vaccinated. Also note that in the next step we get the starting prop. of vaccinated of S_0, γ, by dividing $\mathcal{F}_v$ by S_0.

$$S_{\infty,1} = f^{-1}(I_0 = I_0, \gamma = \frac{\mathcal{F}_v}{S_0}, \mathcal{R}_0 = \mathcal{R}_{0,w}, \phi_S = \phi_{S,1}, q = q)$$
$$S_{T,\infty,1} = \gamma S_0 \big(\frac{S_{\infty,1}}{(1-\gamma)S_0}\big)^{\phi_{S,1}}$$
$$\mathcal{F}_1 = 1 - S_{\infty,1} - S_{T,\infty,1}$$

Note that if $\mathcal{R}_{0,v} = 0$ then $\mathcal{F}_0 = \mathcal{F}_1$.

C.5.3 $\mathcal{F}_{0,vax}$ and $\mathcal{F}_{1,vax}$ for Estimator of Vaccine Causal Effect, Δ_2

For the estimand Δ_2, which calculates the risk difference between the initially vaccinated populations of the trad. vax and trans. vax arms, we first calculate $\mathcal{F}_{0,vax}$, the proportion of infected individuals among initially vaccinated animals in the traditional vaccine arm. We calculate $S_{\infty,0}$ and $S_{T,\infty,0}$ as in Section C.5.2 for Δ_1, then we calculate:

$$\mathcal{F}_{0,vax} = \alpha - S_{T,\infty,0}$$

Which is the initial vaccinated prop. – the prop. still vaccinated after the outbreak. We then calculate $\mathcal{F}_{1,vax}$, the proportion of infected individuals among initially vaccinated animals in the transmissible vaccine arm. We first calculate $\mathcal{F}_v, S_{\infty,1}$, and $S_{T,\infty,1}$ as in Section C.5.2 for Δ_1. Then we calculate:

$$\mathcal{F}_{1,vax} = \mathcal{F}_v - S_{T,\infty,1}$$

Which is the final size proportion among vaccinated individuals (either directly or indirectly vaccinated).

References

[1] Nuismer SL, May R, Basinski A, Remien CH. Controlling epidemics with transmissible vaccines. PLoS One. 2018;13(5):e0196978.

[2] Okwor EC, Eze DC, Umeh M. Newcastle disease virus shedding among healthy commercial chickens and its epidemiological importance. 2011.

[3] Brown VR, Bevins SN. A review of virulent Newcastle disease viruses in the United States and the role of wild birds in viral persistence and spread. Veterinary research. 2017;48:1-15.

[4] Miller PJ, King DJ, Afonso CL, Suarez DL. Antigenic differences among Newcastle disease virus strains of different genotypes used in vaccine formulation affect viral shedding after a virulent challenge. Vaccine. 2007;25(41):7238-46.

[5] Miller JC, Ting T. Eon (epidemics on networks): a fast, flexible python package for simulation, analytic approximation, and analysis of epidemics on networks. arXiv preprint arXiv:200102436. 2020.

[6] Hudgens MG, Halloran ME. Toward causal inference with interference. Journal of the American Statistical Association. 2008;103(482):832-42.

[7] Tsun A. Probability & Statistics with Applications to Computing. Stanford University; 2021.

[8] Walpole RE, Myers RH, Myers SL, Ye K. Probability and statistics for engineers and scientists. vol. 5. Macmillan New York; 1993.

[9] Richiardi L, Bellocco R, Zugna D. Mediation analysis in epidemiology: methods, interpretation and bias. International journal of epidemiology. 2013;42(5):1511-9.

[10] Imai K, Keele L, Tingley D. A general approach to causal mediation analysis. Psychological methods. 2010;15(4):309.

[11] Sheen JK, Haushofer J, Metcalf CJE, Kennedy-Shaffer L. The required size of cluster randomized trials of nonpharmaceutical interventions in epidemic settings. Statistics in Medicine. 2022;41(13):2466-82.

[12] Arino J, Brauer F, van den Driessche P, Watmough J, Wu J. A final size relation for epidemic models. Mathematical biosciences and engineering. 2007;4(2):159.
